# Audiovisual Interactions Among Near-Threshold Oscillating Stimuli in the Far Periphery Are Phase-Dependent

**DOI:** 10.3389/fnhum.2021.642341

**Published:** 2021-08-30

**Authors:** Isma Zulfiqar, Michelle Moerel, Agustin Lage-Castellanos, Elia Formisano, Peter De Weerd

**Affiliations:** ^1^Department of Cognitive Neuroscience, Faculty of Psychology and Neuroscience, Maastricht University, Maastricht, Netherlands; ^2^Maastricht Centre for Systems Biology, Maastricht University, Maastricht, Netherlands; ^3^Maastricht Brain Imaging Centre (MBIC), Maastricht, Netherlands

**Keywords:** multisensory interactions, audiovisual, far periphery, temporal modulation, oscillatory phase

## Abstract

Recent studies have highlighted the possible contributions of direct connectivity between early sensory cortices to audiovisual integration. Anatomical connections between the early auditory and visual cortices are concentrated in visual sites representing the peripheral field of view. Here, we aimed to engage early sensory interactive pathways with simple, far-peripheral audiovisual stimuli (auditory noise and visual gratings). Using a modulation detection task in one modality performed at an 84% correct threshold level, we investigated multisensory interactions by simultaneously presenting weak stimuli from the other modality in which the temporal modulation was barely-detectable (at 55 and 65% correct detection performance). Furthermore, we manipulated the temporal congruence between the cross-sensory streams. We found evidence for an influence of barely-detectable visual stimuli on the response times for auditory stimuli, but not for the reverse effect. These visual-to-auditory influences only occurred for specific phase-differences (at onset) between the modulated audiovisual stimuli. We discuss our findings in the light of a possible role of direct interactions between early visual and auditory areas, along with contributions from the higher-order association cortex. In sum, our results extend the behavioral evidence of audio-visual processing to the far periphery, and suggest – within this specific experimental setting – an asymmetry between the auditory influence on visual processing and the visual influence on auditory processing.

## Introduction

Multisensory information is ubiquitous in our environment. Our brain is adept at pooling information from multiple modalities to form a unified view of our surroundings, thus guiding perception and behavior. The relationship between sensory stimuli (e.g., spatial, temporal, contextual, attentional, etc.) and the task at hand (Spence, 2013; Odegaard and Shams, 2016) helps unify or disassociate binding between senses, leading to changes in behavior [as indexed by discriminability, response times, accuracy, etc. (Chen and Vroomen, 2013; Odegaard et al., 2015, 2016; Bizley et al., 2016)]. These cross-modal interactions can also affect subsequent unisensory processing (Wozny and Shams, 2011; Barakat et al., 2015).

Traditionally, multisensory anatomical and functional processing pathways in human and non-human primates have been credited to converging inputs in higher-order association cortex (Ghazanfar and Schroeder, 2006; Cappe et al., 2009). More specifically, previous studies have shown that audiovisual (AV) integration regions include the posterior superior temporal sulcus and middle temporal gyrus (Beauchamp et al., 2004; van Atteveldt et al., 2004; Tanabe et al., 2005; von Kriegstein et al., 2005; Perrodin et al., 2014; Starke et al., 2017). The intraparietal sulcus (Lewis and van Essen, 2000; Cate et al., 2009) and frontal areas (Gaffan and Harrison, 1991; Romanski et al., 1999; Li et al., 2010) have also been implicated in AV integration.

Early sensory areas, however, have also been shown to play a role in multisensory processing (Hackett et al., 2007; Driver and Noesselt, 2008; Koelewijn et al., 2010). Through the use of anterograde and retrograde tracers, Falchier et al. (2002) showed direct projections from primary and secondary auditory areas to the early visual areas in rhesus monkeys as well as reciprocal connections from secondary visual area (V2) and prostriata to the auditory cortex (Falchier et al., 2010). Evidence for a role of these early cortico-cortical connections in multisensory effects has been functionally established as well. Auditory influences on primary visual areas (V1) have been shown across species (Wang et al., 2008; Ibrahim et al., 2016). The responses in the auditory areas are also directly influenced by the visual cortex (Besle et al., 2008, 2009), for example through changes in the phase of auditory local field potentials and single unit activity (Kayser et al., 2008, 2010). These changes in local field potentials have been shown to amplify sensory inputs (Schroeder and Lakatos, 2009) and, more recently, to provide cross-modal cues in auditory scene analysis (Atilgan et al., 2018). The early onset of the observed multisensory effects supports a role of early sensory cortical connectivity in multisensory interactions (Besle et al., 2008; Wang et al., 2008).

Interestingly, the direct connections between early visual and auditory cortices are not uniform. Instead, neurons with peripheral receptive fields (>30° visual angle) receive and project the majority of these connections (Falchier et al., 2002, 2010; Rockland and Ojima, 2003; Eckert et al., 2008). In accordance, recent human neuroimaging and behavioral studies showed that AV integration varies as a function of space. Some studies showed enhanced AV integration in the periphery compared to foveal locations. For example, this disparity has been reported for the sound-induced flash illusion. The sound-induced flash illusion is a phenomenon where due to audiovisual interactions, two consecutive beeps split a single flash to be perceived as two (fission) or a single beep with the two flashes causes perception of single flash (fusion). The double flash (fission) illusion (induced by sound) dominated behaviorally in the periphery (10° eccentricity) compared to foveally (Chen et al., 2017), and showed stronger activation in the peripheral visual cortex compared to the foveal regions using fMRI (Zhang and Chen, 2006). On the other hand, reduced AV integration in the periphery has also been shown. Specifically, the ventriloquist effect (a shift in the perceived auditory location due to a visual cue) was reported to reduce with increases of eccentricity up to 75° (Charbonneau et al., 2013), and similarly, the fusion illusion was larger in central compared to peripheral (10°) locations (Chen et al., 2017). Thus, while these studies all support that AV integration varies over space, the direction of this variation seems to depend on the experimental task and setup.

In addition to the influence of spatial location, AV integration also depends on the temporal characteristics (Chen and Vroomen, 2013) and salience of the stimuli (Meredith and Stein, 1983; Stein and Stanford, 2008; Stein et al., 2009). To understand how the brain uses temporal features in integrating information from multiple sources, one key approach has been to manipulate the temporal congruency between both naturalistic (McGurk and MacDonald, 1976) and artificial oscillating stimuli (Laing et al., 2015). The temporal characteristics of the stimuli and their salience have also been observed to interact and collectively affect AV integration. In a recent study, the lowest contrast detection thresholds for oscillating visual stimuli were observed when accompanied by in-phase auditory stimuli of weak salience (Chow et al., 2020). While the effects of different stimulus features on audiovisual integration have been extensively studied for more centrally presented stimuli [within 6° visual angle (Shams et al., 2000, 2002; Frassinetti et al., 2002; Spence and Squire, 2003; Soto-Faraco et al., 2004; Kayser et al., 2008; Wu et al., 2009; Chen and Vroomen, 2013; Denison et al., 2013; ten Oever et al., 2014)], the influence of these audiovisual stimulus features on multisensory integration at far-peripheral locations [beyond the 10° visual angle used by Chen et al. (2017) and Chow et al. (2020)] is largely unknown. Therefore, in the present study, we aimed to find the optimal manipulations of temporal features and salience to enhance the multisensory benefit between the far peripheral AV stimuli. To that end, we used a modulation detection task (performed separately for either auditory or visual stimuli) where the stimulus of the other (unattended) modality was always spatially collocated, but presented in either temporal congruence (both modulated and both static) or incongruence (one modulated and the other static). For the other (unattended) modality, weak static and modulated stimuli were used at two different intensities for which preceding measurements had established the temporal modulation to be only barely detectable (corresponding to 55 or 65% correct detection thresholds).

Based on the above review of the literature, we formulated two hypotheses. First, considering the strong involvement of large visual eccentricities in the direct anatomical connectivity linking early visual and auditory areas, we hypothesized that presenting the AV stimuli in spatial congruence at a far-peripheral location would create conditions for strong bidirectional multisensory interactions. Note that we only aimed at testing the multisensory interactions at far-peripheral locations. A direct comparison between far-peripheral and central conditions was beyond the scope of the present study.

Second, we hypothesized that temporal features of the AV stimuli can be manipulated to identify the optimal conditions for both auditory-to-visual and visual-to-auditory interactions. Regarding the second hypothesis, we had two specific predictions. First, we expected that temporal congruence (incongruence) would facilitate (degrade) cross-modal influences in the far periphery. Second, for multisensory interactions in the congruent condition presenting modulated stimuli in both modalities, we expected that the strength of interactions would be phase-dependent, and that interactions could be optimized at appropriate phase relationships. We speculated that any differences in the multisensory interactions due to the small temporal shifts induced by the relative phases at the onset, might be informative in relating the observed responses to the direct interactions between early auditory and visual areas.

## Materials and Methods

### Participants

All twenty-seven participants (mean age 22.8 ± 3.2, including 8 males) had normal or corrected-to-normal vision. A pure-tone audiogram was obtained before the first session to exclude participants with hearing loss (using 25 dB hearing level as a threshold). Prior to the first session, each participant was informed about the procedures, and verbal and written consent was obtained. Participants were compensated with either monetary reward alone or a combination of monetary reward and credit for their course requirements. Following the last session, all participants were debriefed about the purpose of the experiment, and they filled out a questionnaire about their impression of low-intensity stimuli of the unattended modality presented during the tasks. The experiment was approved by the Ethics Review Committee of the Faculty of Psychology and Neuroscience at Maastricht University.

### Apparatus

Participants sat in a soundproof, dimly lit room with their heads supported by a chin and head rest affixed 42 cm in front of a gamma-calibrated LCD monitor (24” Iiyama Prolite B2481HS LED monitor, Iiyama Corporation, Tokyo, Japan; 60 Hz refresh rate, 1,920 × 1,080 resolution). Fixation during the task was checked using ViewPoint Eyetracker (MIU03 Monocular, Arrington Research, Inc., Scottsdale, AZ, United States; 220 Hz) which was mounted toward the left side of the chin and head rest. All stimuli were generated at runtime in MATLAB (The MathWorks, Inc.) using Psychophysics Toolbox (Brainard, 1997; Pelli, 1997; Kleiner et al., 2007). The stimulus PC interfaced with the Eyetracker PC via an Ethernet connection using the ViewPoint Client App and ViewPoint MATLAB toolbox (v2.8.5), providing runtime access to the Eyetracker data.

### Stimuli

The static visual stimulus consisted of a circular sinusoidal grating (vertical orientation, 1.6 grating cycles/degree at a screen resolution of 1,920 pixels × 1,080 pixels, diameter = 6.2°) that was presented at 28.5° eccentricity to the right on the azimuthal plane. A lower modulation rate [3 Hz, prevalent in speech (Overath et al., 2015)] was chosen, in line with previous research where modulated stimuli have been used to study AV interactions in the brain (Laing et al., 2015).

The static sound (Supplementary Figure 1A) was created as a normally distributed white noise stimulus (generated using *randn* in MATLAB with mean = 0, std = 0.5, sampling rate 44.1 kHz). To create the modulated sound (Supplementary Figure 1B), the sound pressure level [SPL, indicated by ‘amplitude’ in Supplementary Figure 1B)] of the static sound (central SPL fixed at ∼32.2 dB) was varied sinusoidally at 3 Hz with a modulation depth of 80%. Sounds were presented using a headphone set (AKG K72). Sound location was matched to the visual stimulus location by adjusting the sounds’ interaural level difference (ILD). ILD was set based on subjective measurements from authors IZ and PDW, and confirmed by each participant at the beginning of the experiment. The resulting ILD of 3 dB is slightly smaller than expected (Shaw, 1974). This difference between our subjective measurements and values reported in the literature may be explained by the overall low intensity of the employed sounds. The intensity of the static stimulus was set at the peak intensity of the corresponding modulated stimulus.

To create the modulated visual stimulus (see Supplementary Figure 1B for reference), the Michelson contrast of the static grating was sinusoidally modulated over time at 3 Hz with a modulation depth of 80%. The contrast of the static stimulus was set at the peak contrast of the corresponding modulated stimulus (see Supplementary Figure 1A for reference).

### Experimental Design

The following sections provide a summary of the experimental conditions. We also detail the staircase design employed in the experiment along with the measurements taken during each session, and the specifications of the task.

#### Summary of Experimental Conditions

**FIGURE 1:**
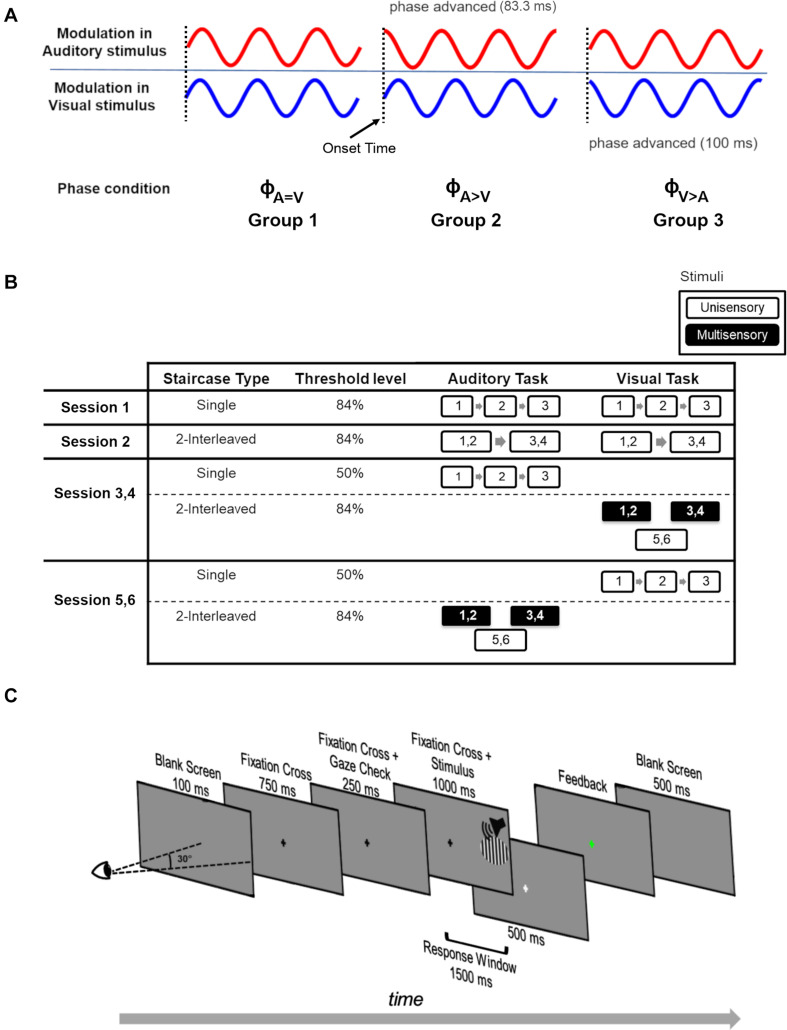
Experimental design. **(A)** The phase of sinusoidally modulated stimuli across the three phase conditions for the three participant groups. For phase condition ϕ_A=V_ (Group 1), the modulated stimuli have no phase-shift. For phase condition ϕ_A>V_ (Group 2), the phase of the modulated auditory stimulus was phase-advanced by 83.3 ms. For phase condition ϕ_A>V_ (Group 3), the modulated visual stimulus was phase-advanced by 100 ms. The onset time of all stimuli is the same (vertical dotted lines). **(B)** Experimental design of the study. Over six sessions (each session conducted on a separate day), participants performed a modulation detection task in a staircase design. Each executed staircase is represented by a numbered box. The number indicates the staircase number, and box size corresponds to the staircase duration. Each staircase measurement resulted in a modulation detection threshold. Depending on the staircase settings, either a 50 or 84% modulation detection threshold was measured (‘Threshold level’). White outlined and black filled boxes represent unisensory and multisensory conditions, respectively. In session 1, three repetitions (indicated by numbers 1–2–3) of single 84% detection threshold staircases were performed on unisensory auditory and visual stimuli. In session 2, participants executed 2-interleaved 84% detection threshold staircases, whose longer duration is indicated by larger boxes, twice each for unisensory auditory and visual stimuli. We then used two types of sessions to collect the multisensory data and associated unisensory control data. In the first type (repeated twice, designated Session 3, 4), three unisensory 50% correct staircases for the auditory task were administered followed by three 2-interleaved 84% correct visual staircases. Auditory stimuli were presented in two of these 2-interleaved 84% correct visual staircases (i.e., they were multisensory), and these staircases were used to measure the influence of auditory stimuli on performance in the visual task. In the second session type (repeated twice, designated Session 5, 6), three unisensory 50% correct staircases for the visual task were administered followed by three 2-interleaved 84% correct auditory staircases. Visual stimuli were presented in two of these 2-interleaved 84% correct auditory staircases (i.e., they were multisensory), and these staircases were used to measure the influence of visual stimuli on performance in the auditory task. Note that the order of the days for sessions 3–6 was randomized over participants. In addition, for sessions 3–6, the order of the 2-interleaved staircases (two multisensory and one unisensory) was varied over participants, but kept the same for an individual participant. **(C)** Experimental design of a single trial. Participants fixated on the fixation cross at the center of the screen. The stimulus was only presented if the participant maintained fixation (fixation error < 2.5°) during the 250 ms gaze check prior to the stimulus presentation. The stimulus was auditory (white noise burst), visual (vertical grating, 6.2° in diameter) or audiovisual, and was presented at 28.5° azimuth in the participants’ right hemifield. The task of the participants was to indicate whether the attended stimulus was modulated or static. Feedback was given by a change in the color of the fixation cross (green = correct, red = incorrect).

The key features of the experimental design are shown in Figure 1. The participants were divided into three groups. Each of these groups took part in one of three conditions (*N* = 9 per condition) that differed from each other in the onset phase (ϕ) of the modulations in the auditory and visual stimuli (Figure 1A). There was no difference in stimulus onset times. For each participant, the experiment consisted of six sessions of 2-h duration each, divided over 6 days (Figure 1B). We used a staircase experimental design to measure the modulation detection thresholds for the visual and auditory stimuli in either a unisensory or a multisensory setting. Within each session, we used a two-alternative forced choice task where participants had to indicate by a button press if a visual or auditory stimulus was modulated or static (Figure 1C). In the multisensory condition, the stimuli were presented in congruent (both modulated or both static) and incongruent (one modulated while other is static) manner. Apart from detection thresholds, response times of the participants were also recorded during the staircases.

In Group 1 (nine participants), the auditory and visual stimuli were modulated sinusoidally (both starting with the default onset phase of a sinusoid being 0). This condition will be referred to as the ϕ_A=V_ condition. In Group 2 (nine participants), the auditory stimulus modulation started with an advanced phase of $\frac{\pi }{2}$ (83.3 ms) while the visual stimulus modulation started at default onset phase of 0 (ϕ_A>V_ condition). In Group 3 (nine participants), the modulated visual stimulus was phase-advanced by $1.2\frac{\pi }{2}$ (100 ms), hence leading in phase compared to the modulated auditory stimulus with no phase-shift at onset. This condition will be referred to as the ϕ_A>V_ condition. All static stimuli remained the same across the three phase conditions. Throughout this manuscript, the term “threshold” refers to the modulation detection threshold (i.e., the stimulus intensity at which participants can discriminate modulated from static stimuli). The term “modulation” always refers to the oscillatory feature of the stimuli rather than to a cross-sensory influence. An overview of all the experimental conditions is provided in Table 1.

**TABLE 1:** Overview of the experimental conditions.

Factor	Task	Task Stimulus Dynamics	Phase Condition	Sensory Condition (Stimulus Dynamics of Other Modality)			Intensity of Influence from Other Modality
				Unisensory	Multisensory		
					Congruent	Incongruent	
**Levels**	Auditory (Visual)	Modulated (different phase per group)	ϕ_A=V_ (Group 1) ϕ_A>V_ (Group 2) ϕ_A>V_ (Group 3)	No stimulus of other modality	Modulated (different phase per group)	Static	55%
							65%
		Static	Same across all three groups		Static	Modulated (different phase per group)	55%
							65%

*The participants performed auditory and visual modulation detection tasks where they indicated the stimulus dynamics (modulated or static) with a button press in a staircase design. Both tasks were performed in unisensory conditions (i.e., the other modality is not present during stimulus presentation) and multisensory conditions. In the multisensory conditions, any given trial presented either a static or a modulated stimulus from one modality together with either a static or modulated stimulus from the other modality. Furthermore, during the multisensory conditions, the AV stimuli were presented in a congruent condition (both static or both modulated) or an incongruent condition (one static, the other modulated). During AV stimulus presentation, the stimulus modality focused on was tested with a staircase converging on 84% correct, while a weak influence from the other modality was presented at an estimated 55 and 65% correct modulation detection threshold determined in separate tests. We had 27 participants in the study who all performed the auditory and visual modulation detection tasks in the two unisensory, and all multisensory conditions, whereby the multisensory conditions presented the weak influence from the other modality at both 55 and 65% levels. However, the 27 participants were subdivided into three groups of 9 participants, each characterized by a different configuration of the starting phases of the modulated stimuli. That is, in Group 1, the onset of both auditory and visual sinusoidally modulated stimuli was affixed at zero (ϕ_A = V_). In Group 2, the auditory stimulus modulation started with an advanced phase of (83.3 ms) while the visual stimulus modulation started at the default onset phase of 0 (ϕ_A > V_). In Group 3, the visual stimulus modulation was phase-advanced by (100 ms), hence leading in phase compared to the modulated auditory stimulus which started at phase zero (ϕ_V>A_). Note that these phase conditions in the three groups applied both to the unisensory and the multisensory conditions.*

#### Staircase Design

We used separate staircases to measure the detection thresholds for modulations in the visual and auditory stimuli. In each staircase, the presentation order of modulated and static stimuli was randomized while ensuring an equal number of modulated and static stimuli for each block of 10 trials.

Three different staircase designs were used during the experiment. To measure 84% detection thresholds, the intensity of the auditory stimulus (contrast of the visual stimulus) decreased for every four consecutive correct answers and increased for every incorrect response (Wetherill and Levitt, 1965). Note that in the staircase measurements, participants had to report the presence or absence of the temporal modulation (modulation depth fixed at 80%) in the stimulus, and that the auditory amplitude or visual contrast were made dependent on performance. This means that for increasingly lower and more challenging steps in the staircase (Supplementary Figure 2), the maximum amplitude of the auditory stimuli, and peak contrast for the visual stimuli, decreased (see also Supplementary Figure 1 for reference). These maximum amplitudes or contrasts also define the static stimuli in, respectively, the auditory and visual modality. Hence, moving to a more challenging step in a staircase meant that a participant (at random) would be presented with either a static stimulus at lower auditory amplitude or visual contrast, or the same stimulus to which the 80% temporal modulation was applied. For staircases with the auditory task, the sound amplitude was varied with 20% for each step. The highest sound amplitude was capped at 37 dB. To compute the contrast steps for the visual staircase, the Michelson contrast was calculated. By fitting a polynomial to the measured contrast values, the luminance values of the screen were converted to corresponding contrast values. The highest contrast value of the grating was capped at 23% Michelson contrast and reduced by a step size of 20%. Each staircase finished either after 14 reversal points were acquired, or upon completion of 120 trials. The 50% auditory (visual) detection thresholds were measured by a staircase where for each wrong/correct response, the auditory intensity (visual contrast) increased/decreased by the respective step size. The 50% detection thresholds converged to stimuli that were so weak that an 80% temporal modulation (i.e., modulation depth) became undetectable. Supplementary Figure 2 shows an example of the staircase procedure used to measure the 84% (Supplementary Figure 2A) and 50% (Supplementary Figure 2B) correct thresholds for a single subject.

We also created interleaved staircases by merging two independent staircases (84% detection threshold), such that trial blocks of two independent staircases were presented in an interleaved fashion. In blocks of 10 trials, the staircase switched pseudo-randomly (to ensure that long stretches of the same staircase did not occur) between the congruent (i.e., auditory and visual stimulus are either static, or both modulated) and incongruent (i.e., one of the multisensory stimuli is static, and the other is modulated) conditions. In order to compare these multisensory conditions to unisensory thresholds, participants also performed unisensory interleaved staircases. Note that the staircases remained independent: responses to trials in one staircase did not affect stimulus presentation in the other staircase. Interleaved staircases finished when both staircases completed either 14 reversal points or 120 trials.

#### Sessions

All participants performed six sessions, which were spread over a period of 2 weeks, with every session at the same time of the day for each participant. Session 1 was designed to familiarize participants with the task, the chin and head rest, and the Eyetracker setup. During session 1, the participants performed three auditory and three visual unisensory staircases in order to determine their 84% modulation detection thresholds. Each staircase took approximately 8 min, and participants were given a break of approximately 5–10 min between staircases (Figure 1B).

In Session 2, the participants completed two interleaved 84% staircases of the unisensory auditory and visual conditions. The duration of an interleaved staircase was approximately 20 min, and participants were given 5–10 min breaks between staircases. The purpose of this session was to provide a baseline behavior for unisensory thresholds in interleaved staircases, as these staircases were then repeated in the next four sessions as discussed below.

Session 3 to 6 started with the estimation of unisensory 50% detection thresholds in three staircases. Next, participants performed two multisensory interleaved staircases. Per session, they performed either the auditory or the visual task, while the static and modulated stimuli from the other modality were shown at a barely-detectable intensity/contrast (allowing an estimated 55 or 65% correct modulation detection threshold). The barely-detectable intensities (SPL for auditory stimuli, contrast for visual stimuli) were selected as they allowed above chance identification of the modulated stimuli yet should not have acted as too strong of a distractor during task execution on the other stimulus modality. The order of these four measurement days [(multisensory auditory or visual task) × (55 or 65%)] was randomized across participants. The 55 and 65% correct modulation detection thresholds were estimated by *z*-scoring the contrast steps between thresholds estimating 84% (from session 2) and 50% correct, and then linearly interpolating the intermediate steps (from 50 to 84%). Supplementary Figure 2C shows an example of how the 65 and 55% correct thresholds were estimated using the two measured thresholds (84 and 50% correct). The resulting psychometric curve is not a straight line because of the conversion from screen luminance to Michelson contrast.

Per session (sessions 3 to 6), two multisensory staircases were conducted. The two interleaved staircases were used to test the effects of (in)congruence of the auditory and visual stimulus on detection thresholds and response times. Participants also performed an interleaved staircase for the unisensory task condition. The order in which participants performed unisensory and multisensory interleaved staircases was balanced across participants to minimize fatigue effects but was kept the same for an individual participant across sessions. Each session took 2 h to complete, and participants were actively encouraged to take breaks during sessions.

#### Task

Participants performed a two-alternative forced choice task on the visual or auditory stimuli. That is, they pressed either the right or left arrow key to report a modulated or static stimulus, respectively (Figure 1C). Each trial began with a gray screen for 100 ms, followed by a black fixation cross that was presented in the center of the screen for 750 ms. For the next 250 ms, the fixation cross remained on the screen while a steady fixation check was performed (with 2.5° freedom from the fixation point). In case of a failed fixation, the trial was aborted, and a new trial began. If the participant passed the gaze check, a stimulus was presented (1 s) while the fixation cross remained on the screen. The response window began at the onset of stimulus presentation and extended 500 ms after stimulus offset (indicated by a light gray fixation cross). Feedback was provided as soon as the participant responded, by a change in the color of the fixation cross to red or green for incorrect and correct responses, respectively. In case the participant did not respond within the response window, the trial condition was appended to the trial list and the next trial was initiated. The inter-trial interval was 500 ms (gray screen). Following every tenth trial, the center for the gaze check was readjusted to correct for drifts of the Eyetracker setup and/or subject motion. The apparatus-induced propagation delay between auditory and visual stimuli was estimated to be ∼20 ms using an oscilloscope with probes on the monitor screen and the headphones.

**FIGURE 2:**
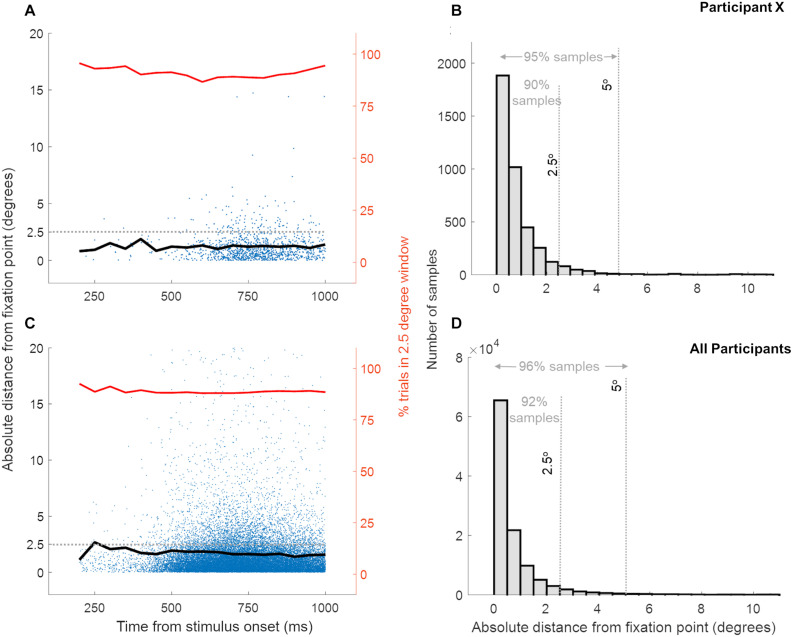
Distance of eye gaze location from the fixation point at the instance of response across trials. Panels **(A,B)** show the data for a single representative participant while panels **(C,D)** show data for all 27 participants, for both multisensory tasks. Each blue dot in panels **(A,C)** represents the eye gaze distance from fixation for a single trial while the black line shows the mean eye gaze distance from fixation over trials. The red line indicates the percentage of trials, at the response time, where the eye gaze location was within 2.5° distance from the fixation point. Panels C and D show the distribution of eye gaze distance from fixation and confirm that eye gaze position was within a few degrees from the fixation point for a high percentage of trials.

In the 250 ms time window before stimulus onset, trials were aborted if gaze position was more than 2.5° away from fixation. Due to a programming error, the eye movements were only recorded in a 5 ms time window before the stimulus presentation (that is if the fixation was maintained in the previous 245 ms), and at the moment of the response. As saccade execution requires ∼200 ms (Şentürk et al., 2016), it is highly likely that participants fixated during the initial part of the stimulus. While we only have eye position recordings at the instance of response, the large number of trials with widely varying response latencies allowed us to sample eye position from ∼250 ms after stimulus presentation until the end of stimulus presentation (1s). Figure 2 shows the distribution of the absolute distance of eye gaze from the fixation point recorded across trials (at the instance of response) during both multisensory tasks (panels A and B show a representative participant; panels C and D show combined data of all participants). Irrespective of the latency of the response (and hence the time since stimulus onset) eye position was within 10° of fixation in 98% of the ∼100,000 samples, within 5° of fixation in 96% of the trials, within 2.5° of fixation in 92% of the trials in 27 participants (Figures 2C,D). Supplementary Figure 3 shows that, across the three participant groups, the eye locations away from fixation did not specifically target the stimulus location. In addition, the data suggest similar fixation performance in the two tasks across the three participant groups. This is supported by a two-way ANOVA over fixation accuracy (the percentage of trials with eye position within 2.5° of the fixation) with between-subject factor “Phase condition” (3 levels: ϕ_A=V_, ϕ_A>V_, ϕ_A>V_) and within-subject factor “Task” (2 levels: Auditory Task – Visual Task) showing neither a significant interaction [*F*(2,24) = 2.43, *p* = 0.1, $\eta_{\text{p}}^{2}$ = 0.1] nor any significant main effects [“Phase Condition”: *F*(2,24) = 0.03, *p* = 0.9, $\eta_{\text{p}}^{2}$ = 3.3e-07; “Task”: *F*(1,24) = 0.1, *p* = 0.7, $\eta_{\text{p}}^{2}$ = 0.004]. Altogether, this evidence supports consistent fixation in our participants and suggests that large fixation errors were present only in a very small minority of trials.

### Statistical Analysis

Modulation detection thresholds for congruent, incongruent, and unisensory stimuli were computed as the average intensity/contrast of the last 10 reversal points and were averaged over repeated staircases. The response times were computed based on all trials spanning the last 10 reversal points and were averaged over repeated staircases as well. Mixed ANOVA analyses (Caplette, 2017), conducted in MATLAB, were used to test for changes in response time and modulation detection thresholds across the three phase conditions, with auditory and visual stimulation, and as driven by the congruency and intensity of multisensory stimulation. After observing significant interactions, we performed follow-up analyses per level of one of the interacting factors while correcting the *F*-ratio of these follow-up analyses by using error term and degree of freedom of significant interaction error term (indicated as *F*_α−*corrected*_) (Hedayat and Kirk, 2006). Bonferroni-corrected pairwise comparison testing was used to further evaluate significant main effects. The effect sizes for ANOVA are reported using partial eta-squared ($\eta_{\text{p}}^{2}$), and Cohen’s *d* (*d*) for pairwise comparisons. The $\eta_{\text{p}}^{2}$ for within-subject ANOVA factors was corrected by the between-subject error sum of squares.

## Results

In the following section, we report the results addressing our two main hypotheses. Our first hypothesis stated that the far-peripheral colocation of the AV stimuli would enable strong bidirectional multisensory interactions. Thus, we presented both auditory and visual stimuli at 28.5° to the right and tested for the presence of the hypothesized multisensory interactions in both visual and auditory modulation detection tasks (using the experimental factor “Task,” see Table 1 for details on all experimental factors). In both tasks, we expected shorter response times and/or ability to correctly detect the modulations at lower intensities due to facilitatory interactions between AV stimuli.

Our second hypothesis involved testing the effects of temporal features of the AV stimuli to determine the best temporal conditions for auditory-to-visual and visual-to-auditory interactions. To that effect, the “Task” (Table 1) required detection of “Stimulus Dynamics” of the presented stimulus (i.e., modulated or static, indicated by a button press) to reach 84% correct modulation detection threshold in a staircase design. During both auditory and visual modulation detection tasks, multisensory conditions (congruent and incongruent) were generated by manipulating “Stimulus Dynamics of the Other Modality.” Furthermore, the stimuli in the unattended modality were presented at two barely-detectable intensities (at an estimated 55 or 65% correct level of modulation detection). In the unisensory condition, the visual and auditory tasks were performed in the absence of stimuli from the other modality. A first expectation was that congruent stimuli would show stronger multisensory interactions than incongruent stimuli.

Our second expectation was focused on the condition in which both the auditory and visual components of the multisensory stimulus were temporally modulated. Specifically, we expected that multisensory interactions might be strongest at a specific phase relationship at the onset of the modulated stimuli, and that these phase relationships might be informative with respect to the contributions of direct pathways between low-level auditory and visual areas in multisensory interactions. Accordingly, the modulated AV streams were presented in three relative “Phase Conditions” (ϕ_A=V_, ϕ_A>V_, ϕ_A>V_; see Figure 1A and Table 1), each of which was tested in a different participant group.

Overall, in all tasks, the effects of “Stimulus Dynamics,” “Sensory Condition,” and “Intensity of Influence from Other Modality” were tested both on modulation detection thresholds and response times (for both correct and incorrect trials). These experimental manipulations did not yield any effects on the modulation detection thresholds (see Supplementary Data 1.1 and Supplementary Figure 4). The response times, however, revealed interesting data patterns, which are presented below. Before reporting on the effects of congruency and the expected phase-dependency of congruent modulated AV stimuli, we also report on unisensory effects driven by stimulus dynamics and phase of modulated stimuli for which we did not originally formulate hypotheses.

At the end of the experiment, participants reported on their perception of the barely-detectable stimulus of the other modality during the auditory and the visual task performed on AV stimuli. Overall, 10 out of 27 participants reported they were unaware of the weak static and modulated stimuli in the to-be-ignored modality. Others reported being moderately (12 out of 27 participants) to largely aware (5 out of 27 participants) of the stimuli in the to-be-ignored modality. From these 17 participants, eight further highlighted that they primarily noticed the presence of the low-intensity visual stimulus (only at the estimated 65% detection threshold intensity) during the auditory task but not vice versa. This suggests that the perceptual quality of visual and auditory stimuli at the same 65% detection level was not equal, and that the visual influences during auditory task execution had the perceptual qualities to capture attention and to be processed/integrated, whereas this was not the case for the auditory influences presented during the visual task. None of the participants reported being aware of (in)congruence between the AV streams.

### Faster Unisensory Response Times for Modulated Than Static Stimuli

**FIGURE 3:**
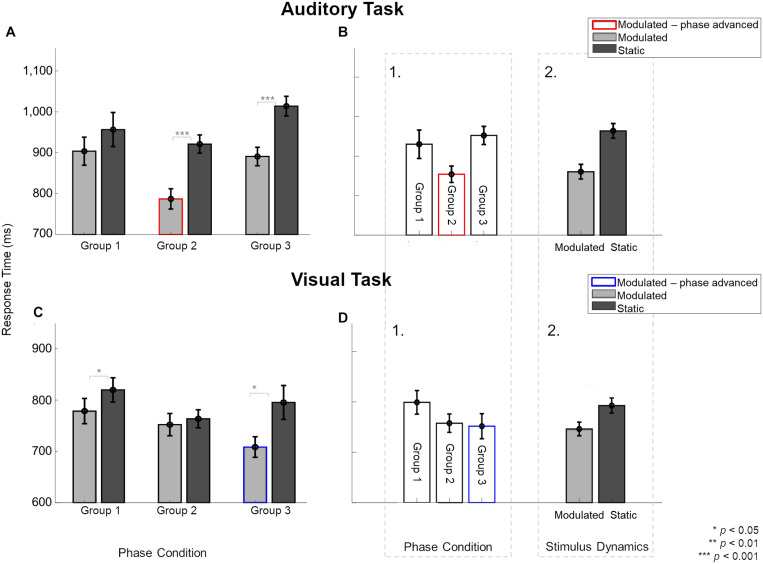
Effect of modulating unisensory stimuli on response times in modulation detection tasks across the three participant groups, averaged over two sessions. Light gray and dark gray bars indicate response times for modulated and static stimuli, respectively. Red and blue borders indicate the modulation conditions with phase shifts. **(A)** In the auditory task, participants in Group 2 and 3, but not Group 1, were significantly faster to identify modulated than static sounds. Response times to auditory stimuli not only varied between the three groups **(B1)** but also for stimulus dynamics **(B2)**. **(C)** In the visual task, participants in Group 1 and 3, but not Group 2, were significantly faster to identify modulated than static sounds. **(D1)** Group had no overall effect on response times. Only stimulus dynamics **(D2)** significantly affected the response times for visual stimuli. Error bars indicate ±1 SEM.

The 84% unisensory measurements (auditory and visual) were made during three sessions, i.e., Session 2, and then twice during Sessions 3 – 6. We studied the effects of temporal feature manipulation (through changing stimulus dynamics and manipulating the phase of the modulated stimuli at onset) on unisensory response times using only the unisensory data collected during Sessions 3 – 6. Figure 3 shows unisensory response times measured during auditory (A–B) and visual (C–D) tasks. In the context of unisensory stimuli, the labels refer to the assignment of the three participant groups to the phase-related manipulation of the unimodal modulated visual (Group 1 and Group 2 have no phase-shift, Group 3 is phase advanced and indicated by blue outline) or unimodal modulated auditory (Group 1 and Group 3 have no phase-shift, Group 2 is phase advanced and indicated by red outline) stimuli. Static stimuli are the same across groups. Figures 3A,B show the response times (averaged across the two sessions) during the unisensory auditory task for the two main stimulus dynamics conditions (static vs. modulated) split over the three participant groups.

To analyze the unisensory auditory response times, a three-way ANOVA was performed in which participants were assigned to the same conditions as used for the multisensory part of the experiments. Hence, the unisensory data were analyzed to test effects of the “Phase condition” (3 levels: Group 1 – Group 2 – Group 3), as well as the “Stimulus dynamics” (2 levels: modulated – static) and “Session” (2 levels: unisensory measurements from the two sessions). The three-way interaction [*F*(2,24) = 2.49, *p* = 0.1, $\eta_{\text{p}}^{2}$ = 0.172], and the two-way interaction between “Phase condition” and “Session” [*F*(2,24) = 0.46, *p* = 0.6, $\eta_{\text{p}}^{2}$ = 0.03] were not significant. There were, however, two significant two-way interactions between the factors “Stimulus dynamics” and “Session” [*F*(1,24) = 5.45, *p* = 0.02, $\eta_{\text{p}}^{2}$ = 0.185], and “Phase condition” and “Stimulus dynamics” [*F*(2,24) = 4.79, *p* = 0.01, $\eta_{\text{p}}^{2}$ = 0.286]. The significant two-way interaction between “Stimulus dynamics” and “Session” was further explored with a pairwise comparison between modulated and static sounds per session. These analyses showed that responses to modulated stimuli were faster than to static sounds in both sessions {55%: *t*(8) = −8.06, *p*[corrected] < 0.001, *d* = 1.551; 65%: *t*(8) = −6.49, *p*[corrected] < 0.001, *d* = 1.249}.

The interaction between “Phase condition” and “Stimulus Dynamics” was further explored for each phase condition (Figure 3A). Pairwise comparisons showed that the response times for modulated stimuli were statistically faster than static stimuli in two of three cases {for Group 2: *t*(8) = −5.94, *p*[corrected] = 0.001, *d* = 1.98 and Group 3: *t*(8) = −16.45, *p*[corrected] < 0.001, *d* = 5.484 but not Group 1: *t*(8) = −2.07, *p*[corrected] = 0.2, *d* = 0.691}. Because Group 3 and Group 1, in the context of the unisensory task, have physically identical stimuli, the presence of a statistical difference between static and modulated conditions for Group 3 and not for Group 1 could reflect an influence of the multisensory context in which the unisensory task was embedded, or a group difference. As the order in which unisensory measurements were taken was randomized across subjects (measured before any multisensory exposure in one-third of participants), our data is limited in the ability to shed light on this observed difference.

For unisensory visual response times, a three-way ANOVA with between-subject factor “Phase condition” (3 levels: Group 1 – Group 2 – Group 3) and within-subject factors “Stimulus dynamics” (2 levels: modulated – static) and “Session” (2 levels: unisensory measurements from the two sessions) showed only a significant two-way interaction between “Phase condition” and “Stimulus Dynamics” [*F*(2,24) = 4.94, *p* = 0.015, $\eta_{\text{p}}^{2}$ = 0.292]. The three-way interaction [*F*(2,24) = 0.12, *p* = 0.88, $\eta_{\text{p}}^{2}$ = 0.01] and all other two-way interactions were not significant [“Stimulus Dynamics” and “Session”: *F*(1,24) = 2.49, *p* = 0.67, $\eta_{\text{p}}^{2}$ = 0.007; “Phase condition” and “Session”: *F*(2,24) = 3, *p* = 0.06, $\eta_{\text{p}}^{2}$ = 0.2]. The significant two-way interaction was explored for each phase condition (Figure 3C). The response times for modulated stimuli were faster than static stimuli for Group 1 {*t*(8) = −3.52, *p*[corrected] = 0.02, *d* = 1.173} and Group 3 {*t*(8) = −3.85, *p*[corrected] = 0.01, *d* = 1.286} but not for Group 2 {*t*(8) = −0.74, *p*[corrected] > 0.99, *d* = 0.249}. Here, again the physical conditions for the unisensory task were identical in Group 1 and Group 2 conditions but led to different outcomes of statistical testing.

Figure 3A shows an overall trend for modulated auditory stimuli to yield faster response times (gray bars) compared to static stimulus (dark bars). Additionally, when the modulated stimulus was also phase-advanced (red-outlined gray bar), the response time was fastest. Figure 3B1 visually illustrates that a phase-advanced auditory stimulus provided a response time advantage. Figure 3B2 visually illustrates a sizeable response time advantage for modulated auditory stimuli over static stimuli. Overall, in both tasks, modulated stimuli (gray bars) yielded faster response times than static stimuli, and phase advancing the modulated stimulus (bars with colored outlines) provided an extra response time advantage.

In the task on visual unisensory stimuli (Figures 3C,D), analogous to findings with the auditory unisensory task, the overall trend toward a response time advantage for modulated stimuli appeared to be strengthened when phase advancing the visual modulated stimulus (Figure 3D, blue-outlined bar). Again, there was no physical difference between the stimuli shown in the three groups of participants, except that the modulated visual stimulus was phase-advanced in the ϕ_A>V_ group compared to the modulated stimuli in the two other groups. There was no clear difference in response time among the different groups (Figure 3D1), but as with the auditory task, response times were faster for modulated than static auditory stimuli (Figure 3D2). This shows that also for the visual unisensory task, the main finding is an overall response time advantage for the modulated stimulus, which is strengthened by phase advancing the visual modulated stimulus.

### Phase-Dependent Response Time Reduction in Auditory Task Due to Visual Influences

**FIGURE 4:**
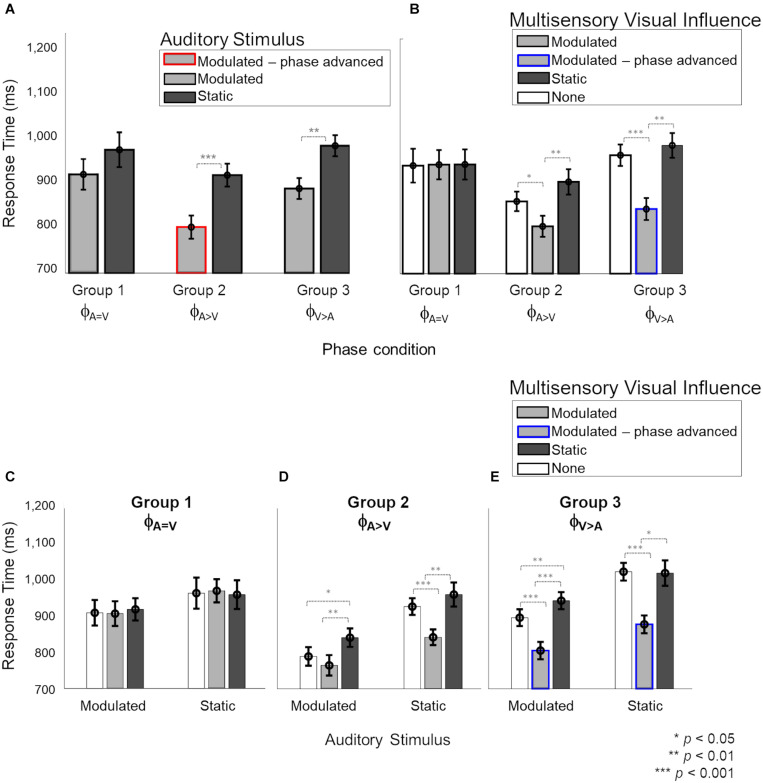
Response times during the auditory task with visual influences. In panels **(B–E)**, light and dark gray bars represent the presence of barely-detectable modulated and static visual influences, respectively, while white bars show the unisensory condition. Red and blue lines indicate phase-advanced auditory and visual conditions, respectively. Error bars represent ±1 SEM. Panels **(A,B)** show the main effects of “Auditory stimulus” (2 levels: modulated – static) and “Visual influence” (3 levels: none – modulated – static), respectively. Panels **(C–E)** the interaction of “Auditory stimulus” and “Visual influence” plotted for separately for phase conditions ϕ_A= V_, ϕ_A>V_ and ϕ_V> A_, respectively. While no effect of the visual influence on the auditory stimulus was observed in phase condition ϕ_A=V_ **(C)**, responses to sounds were significantly faster when a modulated compared to static visual influence was present in phase conditions ϕ_A>V_ and ϕ_V> A_ **(D,E)**. **(C)** For phase condition ϕ_A=V_, when the auditory stimulus and visual influence were in-phase (no phase shift for modulated stimuli), no significant interaction between the auditory stimulus and visual influence was observed. **(D)** For phase condition ϕ_A>V_ (modulation phase of the auditory stimulus was leading with respect to that of the visual influence), we observed an overall distraction effect of the static visual influence and no advantageous effect of the visual influence for modulated auditory stimuli. Response times for the static auditory stimuli became faster due to the modulated visual influence. **(E)** For phase condition ϕ_A>V_ (the modulation phase of the visual influence was leading with respect to that of the auditory stimulus), an advantage of the modulated visual influence, as well as a disadvantage in case of a static visual influence, were observed compared to unisensory sounds.

Next, we evaluated the multisensory influence of weak (static or modulated) stimuli in one modality on the response time for stimuli in the other modality. We focused first on the influence of visual stimuli on responses to auditory stimuli. We were interested in the effect of the different onset phases for the modulated auditory and modulated visual stimuli, and also in the cross-sensory interactions between modulated and static stimuli of the auditory and visual modalities. Figure 4 shows all the effects observed for auditory response times. The different factors contributing to the observations are shown in different panels. In panel A, the response times are grouped by the auditory stimulus (modulated and static) for the three phase conditions. Panel B shows the same response time data as in panel A, but categorized by the different multisensory visual influences (modulated, static or no visual stimulus) during the auditory task. In panels C, D and E, the different effects of visual influence (modulated, static or no visual stimulus) on modulated and static auditory stimuli due to the three phase conditions are shown individually.

A mixed four-way ANOVA of between-subject factor “Phase condition” (3 levels: ϕ_A=V_ – ϕ_A>V_ – ϕ_A>V_) and the three within-subject factors “Auditory stimulus” (2 levels: modulated – static), “Visual influence” (3 levels: modulated – static – none) and “Intensity” of the visual influence (2 levels: 55–65%) showed a significant four-way interaction [*F*(4,48) = 2.957, *p* = 0.029, $\eta_{\text{p}}^{2}$ = 0.198]. The level ‘none’ indicates the absence of a visual stimulus and thus refers to unisensory auditory response time measurements from the two multisensory sessions. Further analysis of the interaction showed that the effect of “Intensity” of the visual influence was not significant (see Supplementary Data 1.2 and Supplementary Figure 5 for details). Thus, to simplify the interpretation of the effects, we averaged over the data for the two intensities before exploring a mixed ANOVA with the between-subject factor “Phase condition” and two within-subject factors: “Auditory stimulus” and “Visual influence.” Results showed a significant three-way interaction [Figure 4, *F*(4,48) = 3.11, *p* = 0.023, $\eta_{\text{p}}^{2}$ = 0.206], which we further explored by analyzing the data per “Phase condition” using a repeated measures ANOVA (with factors “Auditory stimulus” and “Visual influence”). The main effects of “Auditory stimulus” and “Visual influence” on response time for the auditory stimulus are shown separately in Figures 4A,B, respectively, for the three phase conditions (ϕ_A=V_, ϕ_A>V_, ϕ_A>V_). The interaction effect is broken down into effects of visual influence on modulated and static auditory stimuli for the three phase conditions in Figures 4C–E, respectively.

In the ϕ_A=V_ condition (Group 1), Figures 4A,B show that there were no main effects, neither of the factors “Auditory stimulus” {Figure 4A, *F*(1,8) = 6.16, *p*[corrected] = 0.11, $\eta_{\text{p}}^{2}$ = 0.3} nor of “Visual influence” {Figure 4B, *F*(2,16) = 0.03, *p*[corrected] > 0.999, $\eta_{\text{p}}^{2}$ = 0.001}, and the two factors also did not interact [Figure 4C, *F*_0.016_(2,48) = 0.83, *p* = 0.44, $\eta_{\text{p}}^{2}$ = 0.024].

In the ϕ_A>V_ condition (Group 2), the main effects of “Auditory stimulus” {Figure 4A, *F*(1,8) = 28.54, *p*[corrected] < 0.001, $\eta_{\text{p}}^{2}$ = 0.675} and “Visual influence” {Figure 4B, *F*(2,16) = 18.28, *p*[corrected] < 0.001, $\eta_{\text{p}}^{2}$ = 0.547}, and their interaction [Figure 4D, *F*_0.016_(2,48) = 6.11, *p* = 0.004, $\eta_{\text{p}}^{2}$ = 0.189] were significant. The interaction was further explored with a separate one-way ANOVA for modulated and static auditory stimuli. For modulated auditory stimuli (Figure 4D, left), there was a significant effect of the “Visual influence” [*F*_0.008_(2,48) = 19.67, *p* < 0.001, $\eta_{\text{p}}^{2}$ = 0.351]. Pairwise comparisons showed that the presence of a modulated visual influence significantly sped up response times in comparison with a static visual stimulus {compare gray to dark bar, *t*(8) = −4.31, *p*[corrected] = 0.007, *d* = 1.438}, but a modulated visual influence did not give a significant advantage in comparison with the unisensory condition {compare gray to white bar, *t*(8) = −1.78, *p*[corrected] = 0.33, *d* = 0.594}. However, the presence of a static visual influence significantly slowed down response times as compared with a unisensory auditory stimulus {compare white to dark bar, *t*(8) = 3.55, *p*[corrected] = 0.02, *d* = 1.182}. For static auditory stimuli (Figure 4D, right), there was a significant effect of the visual influence as well [*F*_0.008_(2,48) = 47.69, *p* < 0.001, $\eta_{\text{p}}^{2}$ = 0.539]. Responses to static auditory stimuli were faster when accompanied by a modulated, non-phase-advanced visual influence. This was true when comparing to a visual static influence {compare gray to dark bars, *t*(8) = −5.44, *p*[corrected] = 0.001, *d* = 1.812} and when comparing to a situation in which there was no visual influence at all {compare gray with white bar, *t*(8) = −6.13, *p*[corrected] < 0.001, *d* = 2.046}. The response times for the static auditory stimulus were the same irrespective of whether it was paired with a visual static influence or with no visual stimulus at all {compare dark and white bars, *t*(8) = 1.89, *p*[corrected] = 0.28, *d* = 0.63}.

In the ϕ_A>V_ condition (Group 3), the main effects of “Auditory stimulus” {Figure 4A, *F*(1,8) = 87.50, *p*[corrected] < 0.001, $\eta_{\text{p}}^{2}$ = 0.72} and “Visual influence” {Figure 4B, *F*(2,16) = 27.19, *p*[corrected] < 0.001, $\eta_{\text{p}}^{2}$ = 0.68} were significant. There was also a significant interaction between factors “Auditory stimulus” and “Visual influence” [*F*_0.016_(2,48) = 5.94, *p* = 0.005, $\eta_{\text{p}}^{2}$ = 0.14; Figure 4E. Further investigation of the interaction (Figure 4E) showed that the presence of a visual influence significantly changed response times for both modulated and static auditory stimuli [modulated: *F*_0.025_(2,48) = 62.98, *p* < 0.001, $\eta_{\text{p}}^{2}$ = 0.659; static: *F*_0.025_(2,48) = 87.98, *p* < 0.001, $\eta_{\text{p}}^{2}$ = 0.554]. For modulated auditory stimuli (Figure 4E left), there was a response time advantage when there was a modulated rather than a static visual influence {compare gray to dark bars, *t*(8) = −7.7, *p*[corrected] < 0.001, *d* = 2.566}, and also when there was a modulated rather than no visual influence {compare gray to white bars, *t*(8) = −8.55, *p*[corrected] < 0.001, *d* = 2.851}. Response times for the modulated auditory stimulus were slower when there was a visual static influence when compared to absence of visual influence {compare dark and white bars, *t*(8) = 5.77, *p*[corrected] = 0.001, *d* = 1.926}. A similar data pattern was present for response times for static auditory stimuli {Figure 4E right, visual modulated vs. visual static influences: *t*(8) = −3.41, *p*[corrected] = 0.02, *d* = 1.139; visual modulated influences vs. no visual influence at all: *t*(8) = −7.45, *p*[corrected] < 0.001, *d* = 2.485; visual static vs. no visual influence at all: *t*(8) = −0.15, *p*[corrected] > 0.999, *d* = 0.051}.

Overall, in the auditory task, we found no effect of visual influences for the phase-aligned (ϕ_A=V_) condition. We observed that the visual phase-advanced modulatory stimuli (ϕ_A>V_) sped up the detection of static and modulated (not phase-advanced) auditory stimuli (Figure 4E). For a modulated auditory stimulus in phase condition ϕ_A>V_, the presence of a modulated (not phase-advanced) visual influence did not provide an advantage over the unisensory condition (Figure 4D). The static visual stimuli had a distracting effect (increased response times) on modulated auditory stimuli in phase conditions ϕ_A>V_ and ϕ_A>V_ (Figures 4D,E).

### Response Times in Visual Task Do Not Benefit From Auditory Influences

**FIGURE 5:**
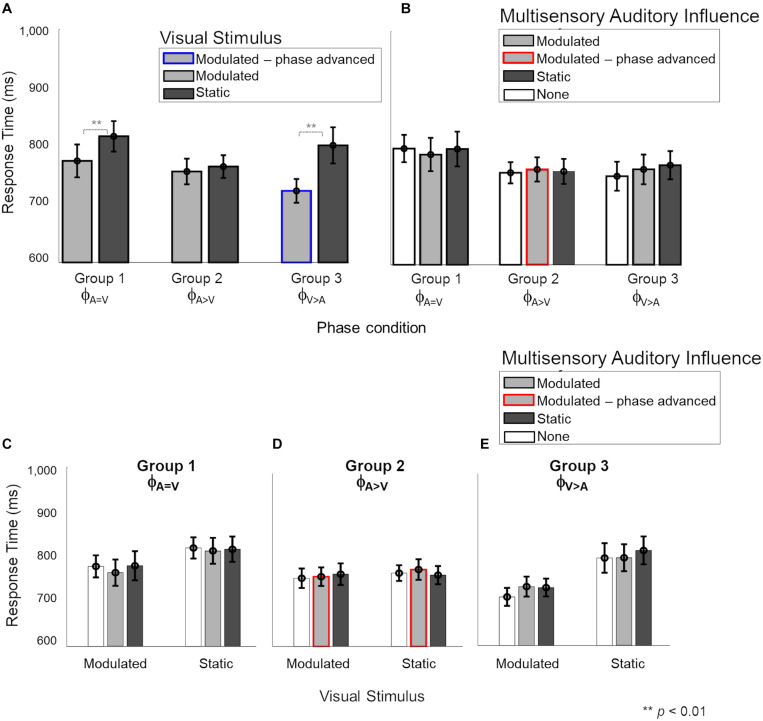
Response times during the multisensory visual task. In panels **(B–E)**, light and dark gray bars show the presence of barely-delectable modulated and static auditory influences, respectively, while white bars show the unisensory condition. Red and blue lines indicate phase-advanced auditory and visual conditions, respectively. **(A)** There was a significant main effect of “Visual stimulus” (2 levels: modulated – static) where responses for modulated stimuli were faster than static stimuli for phase conditions ϕ_A=V_ and ϕ_A>V_. There was no main effect of “Auditory influence” (3 levels: none – modulated – static). **(B)** Nor any significant interaction between the auditory stimulus and visual influence for any phase conditions **(C–E)**. Error bars represent ±1 SEM.

We then explored the reciprocal effect of weak auditory stimuli on response times for visual stimuli (Figure 5). In panels A and B, the response times are grouped by the visual stimulus (modulated and static) and multisensory auditory influence (modulated, static, or no visual stimulus) during the visual task, respectively. Panels C–E show the individual effects of auditory influence on modulated and static visual stimuli in the three phase conditions.

A mixed four-way ANOVA of between-subject factor “Phase condition” (3 levels: ϕ_A=V_ – ϕ_A>V_ – ϕ_A>V_), and the three within-subject factors “Visual stimulus” (2 levels: modulated – static), “Auditory influence” (3 levels: modulated – static – none) and “Intensity” of the auditory influence (2 levels: 55–65%) did not show a significant four-way interaction [*F*(4,48) = 2.44, *p* = 0.059, $\eta_{\text{p}}^{2}$ = 0.169, see Supplementary Figure 6]. However, the three-way interaction between “Phase condition,” “Visual stimulus,” and “Auditory influence” was significant [*F*(4,48) = 2.687, *p* = 0.042, $\eta_{\text{p}}^{2}$ = 0.183], and we therefore further analyzed the data per phase condition.

The effect of “Visual stimulus” (Figure 5A) was significant for the ϕ_A=V_ condition {Group 1, *F*(1,16) = 14.08, *p*[corrected] = 0.015, $\eta_{\text{p}}^{2}$ = 0.46} and the ϕ_A>V_ condition [Group 3, *F*(1,8) = 18.44, *p* = 0.006, $\eta_{\text{p}}^{2}$ = 0.62] but not for the ϕ_A>V_ condition {Group 2, *F*(1,16) = 0.45, *p*[corrected] > 0.999, $\eta_{\text{p}}^{2}$ = 0.029}. The effect of “Auditory influence” (Figure 5B) failed to reach significance for all phase conditions {ϕ_A=V_: *F*(2,16) = 0.51, *p*[corrected] > 0.999, $\eta_{\text{p}}^{2}$ = 0.03; ϕ_A>V_: *F*(2,16) = 0.475, *p*[corrected] > 0.999, $\eta_{\text{p}}^{2}$ = 0.04; ϕ_A>V_: *F*(2,16) = 0.03, *p*[corrected] = 0.05, $\eta_{\text{p}}^{2}$ = 0.14}. There was no significant interaction between factors “Visual stimulus” and “Auditory influence” for ϕ_A=V_ {Figure 5C; *F*(2,16) = 1.86, *p*[corrected] = 0.5, $\eta_{\text{p}}^{2}$ = 0.01}, ϕ_A>V_ {Figure 4D; *F*(2,16) = 1.63, *p*[corrected] = 0.66, $\eta_{\text{p}}^{2}$ = 0.04}, and ϕ_A>V_ {Figure 5E; *F*(2,16) = 2.67, *p*[corrected] = 0.2, $\eta_{\text{p}}^{2}$ = 0.065}.

To summarize, the responses to modulated visual stimuli were faster than to static visual stimuli for ϕ_A=V_ and ϕ_A>V_, as was observed already in the unisensory measurements as well. We observed no effect of low-intensity auditory influences on the response times for visual stimuli, thus confirming the lack of effective auditory influences while performing the visual task.

## Discussion

In the present work, we detailed the effect of auditory-to-visual and visual-to-auditory interactions in the far periphery using simple stimuli (gratings and noise bursts). For both an auditory and a visual task, we studied the influence of multisensory temporal (in)congruence on modulation detection threshold and response time by using static and modulated stimuli and manipulating the relative phase of the modulated AV stimuli. In multisensory conditions, the multisensory influence from the other modality was presented using static and modulated stimuli permitting an estimated threshold of 55% or 65% correct modulation detection. We had originally hypothesized bi-directional cross-sensory effects, but the data only showed visual-to-auditory interactions. The temporal feature manipulation (i.e., temporal modulations and their onset phases) led to interesting observations not just for the multisensory condition but in the unisensory condition as well. Also, the congruency-based facilitation that was expected in the data, showed rather complex patterns dependent on the temporal features of the stimuli. These observations are discussed below.

We report three main sets of findings. First, in the unisensory conditions, we found that the response times were generally faster for modulated stimuli compared to static stimuli for both auditory and visual modalities. We had not hypothesized this finding, but it is in line with the advantage of having a temporal modulation in a peripheral visual stimulus (Hartmann et al., 1979) and with the human sensitivity to temporally structured stimuli in audition (Joris et al., 2004). We also found that advancing the phase of the modulated stimulus to a sharp intensity/contrast change (from maximum to minimum) at the onset of the stimulus, further shortened the response times. The phase advancement creates both a stronger onset and a maximal intensity change from maximal to minimal at the beginning of the stimulus. Our observations hence show the key role of both factors in the detection of modulated stimuli. Overall, visual response times were found to be faster than auditory response times. While generally auditory response times have been reported to be faster than visual response times (Arrighi et al., 2005; Shelton and Kumar, 2010; Ng and Chan, 2012; Jain et al., 2015), the opposite trend has also been observed showing the dependence of this effect on a specific task and stimulus features (Shams et al., 2010).

Second, we observed a cross-sensory effect of visual stimuli on response times for auditory stimuli. In line with our second hypothesis, depending on temporal (in)congruence and synchrony between modulated AV streams, we observed that visual influences could not only speed up (facilitation effects) the response times for auditory stimuli but could also slow them down (degradation effects). We first consider the facilitation effects of modulated visual influences on modulated auditory stimuli during the auditory task (see the left halves of Figures 4C–E). Confirming our second hypothesis, these effects depended on the phase relations between the visual and auditory streams. When the phase of the visual modulation led the modulated sound by 100 ms in the auditory task (ϕ_A>V_), a multisensory benefit (i.e., faster response times for both modulated and static sounds) due to the modulated visual influence was observed (gray bar Figure 4E, left). However, when auditory and visual modulations were in phase (ϕ_A=V_), no multisensory interaction was observed (gray bars Figure 4C, left). This finding of a visually-driven benefit on response time for modulation detection in the peripheral sounds when the concurrent visual stimulus is phase-advanced by 100 ms might indicate a role of the direct influences from early visual to early auditory cortex. The response time advantage cannot be attributed to increased salience at the onset of the phase-advanced visual stimulus, as the static visual stimuli have the same salience at onset yet provide no advantage. Thus, the temporal dynamics of the visual stimulus must play a role. In the phase-advanced visual stimulus, the maximum-to-minimum intensity sweeps in the visual stream precede the analogous intensity sweeps in the auditory stimulus by 100 ms. Taking into account that neuronal response latencies are longer for visual stimuli than sounds [55 ms (Schroeder et al., 2008) in V1, and 23 ms (Besle et al., 2008) in A1], the visual intensity sweeps would have ∼75 ms to carry cross-modal information to the early auditory areas that could facilitate auditory neural activity in response to the auditory sweeps. This is short enough to be compatible with direct interactions between early cortical sites and shows the prominent role of stimulus features at onset in driving the cross-modal advantages. Such early advantages may provide a benefit to multisensory information processing in higher-order cortical regions. Note that when the modulated auditory stimulus itself was phase-advanced ϕ_A>V_, the visual modulated influence did not provide a response time benefit (gray bars Figure 4D, left).

The underlying mechanisms and pathways for the observed multisensory interaction cannot be disentangled based on the present study and would require future neuroimaging and electrophysiological studies. However, the current findings can be put into perspective based on existing evidence of mechanisms that underlie cross-sensory effects. The oscillatory phase of the internal cortical rhythms is known to play a role in auditory perception through interactions with cross-sensory visual and motor cues (Mercier et al., 2015; Simon and Wallace, 2017; Benedetto et al., 2018; Ikumi et al., 2019; Bauer et al., 2020; Mégevand et al., 2020; Thézé et al., 2020; Assaneo et al., 2021). There is even evidence of “oscillatory phase-resetting” in multisensory interactions among early sensory cortices (Lakatos et al., 2007; Doesburg et al., 2008; Schroeder and Lakatos, 2009; Atilgan et al., 2018). More specifically, visual or somatosensory stimuli may influence auditory processing by resetting the phase of ongoing oscillatory auditory cortical activity. Cross-sensory phase-resetting has been observed in early auditory areas with influences coming from somatosensory (Kayser and Kayser, 2018) and visual input (Kayser et al., 2010). Facilitation or suppression effects have been shown to be dependent on the temporal relationship between the onsets of stimuli (Kayser et al., 2010), in line with the lead in onset for visual compared to auditory stimuli in the ϕ_A>V_ condition in the present study. Additionally, these effects are more pronounced at near-threshold levels (Schroeder and Lakatos, 2009; ten Oever et al., 2014) compatible with the low-contrast visual stimuli we have used. Based on our observations for phase condition ϕ_A>V_, where a leading modulated visual stimulus provided a response time benefit to static and modulated sounds, the sharp intensity changes at the first part of the visual stimuli (from peak to trough contrast), as well as the recurring intensity changes in further cycles of the visual oscillation, may have caused phase-resets in local oscillatory activity in the early visual cortices. These changes, in turn, might have led to an enhanced representation of the auditory information, engaging sensory integration between early cortical sites. While its underlying mechanism remains speculative, our results may provide a basis for future experiments.

An additional facilitatory effect of modulated visual influence was observed for static auditory stimuli in the ϕ_A>V_ and ϕ_A>V_ conditions, but not the ϕ_A=V_ condition (gray bars in right-hand parts of Figures 4C–E). The facilitatory effect of the modulated visual influence in the ϕ_A>V_ condition is remarkable because the AV stimuli in that condition and the ϕ_A=V_ condition were identical (i.e., the same static auditory stimulus combined with the same visual influence). Therefore, the advantages in the ϕ_A>V_ condition (and possibly also the ϕ_A>V_ condition) for the static auditory stimulus somehow were acquired indirectly from the advantages experienced by the modulated auditory stimulus from the modulated visual influences, thus implying cross-trial effects. It is not clear how these cross-trial influences occur, but in a broad sense, they are in line with the idea that audiovisual interactions can occur at multiple stages of sensory processing (Cappe et al., 2009; Koelewijn et al., 2010). Hence, while the observed data might show a potential role of the direct visual-to-auditory influences at peripheral locations in multisensory processing, the observed cross-trial dependencies of visual-to-auditory benefits to trials with static stimuli might also rely on contributions of higher association cortices in the brain (Covic et al., 2017). As in our experiment design, the (in)congruent modulated and static stimuli are presented randomly in a staircase design with varying intensity of stimuli, we are unable to comment on the nature of serial interactions extending over trials. Furthermore, if and how these serial interactions affect the responses to modulated stimuli, also remains unknown. Altogether, these observations pose interesting questions for further research.

We also observed a degradation effect of static visual influence. That is, only in the presence of a phase difference between AV streams did the static visual influence slow down the response time for auditory stimuli compared to the unisensory and congruent modulated conditions. This effect, however, was present only for the modulated auditory (Figures 4D,E left) and not for the static auditory stimuli (Figures 4D,E right). Our findings may represent a distraction effect of the static visual stimulus. A possible explanation for the absence of this effect in phase condition ϕ_A=V_ might be found in the overall longer response times for that condition. As participants already took a long time to respond, the presence of the static visual stimulus may not have further slowed the responses down. That is, the static visual stimuli can only provide a disadvantage in the case of a comparative advantage driven by phase-advanced modulated auditory stimuli (ϕ_A>V_) or visual stimuli (ϕ_A>V_). A slightly different view on these degradation effects is the idea that, especially in the cases where the modulated visual influence is integrated with the modulated auditory stimulus (as witnessed by a response time benefit), a static visual influence will be harmful. Hence, the observed degradation effects also support a form of audiovisual interaction.

While we have discussed the multisensory facilitation of the response times primarily in light of the temporal dynamics of the stimuli, the redundant target effect (RTE; Ridgway et al., 2008) should also be considered as a potential mechanism for explaining our findings. The RTE predicts that the response time to AV stimuli is driven by the stimulus with the fastest processing time. As the response time to unisensory visual stimuli is faster than the response to unisensory auditory stimuli, RTE can explain the observed lack of an auditory influence on visual response times. RTE can also attribute the beneficial effect of the modulated visual influence on auditory response times in the phase-shift conditions (Groups 2 and 3) to the faster unisensory response times for modulated visual stimuli. However, beyond these observations, the unisensory response times we observe do not translate to the multisensory response time data pattern, opposing RTE predictions. For example, we observe faster RTs to modulated (compared to static) unisensory visual stimuli in Groups 1 and 3, but not for Group 2 (Figure 3C). This pattern does not fit with the visual influence on auditory response times (Figure 4B), where RTs are faster for modulated (compared to static) stimuli in Groups 2 and 3, but not for Group 1.

Additionally, if driven by RTE, AV response times should always be faster than unisensory auditory response times, as the response to any unisensory visual stimulus is faster than to any unisensory auditory stimulus. Instead, we observed that a static visual influence in the AV conditions resulted in longer response times than observed for unisensory auditory conditions (Groups 2 and 3; Figures 4D,E). These mismatches between patterns in the unisensory response times and the multisensory data patterns argue against the RTE being the driving mechanism for the reported observations.

Lastly, our third main finding was related to the visual task where we found that a weak auditory influence (at an estimated 55 or 65% correct modulation detection threshold) did not affect visual detection thresholds or response times. This finding contradicts our first hypothesis of bidirectional cross-sensory effects between audition and vision. It could be argued that the absence of an effect of weak auditory stimuli on the visual task may have been driven by low power due to insufficient sample size. However, the clear lack of trends tending to significance in the visual task data makes this unlikely. Nevertheless, previous studies have shown auditory influences on responses in the visual cortex (Wang et al., 2008; Bolognini et al., 2010; Ibrahim et al., 2016) and have also shown behavioral (dis)advantages (Di Russo et al., 2002; Shams et al., 2002). Because of these studies, and because the connectivity between early sensory regions is strong in the periphery and provides pathways for influences between early visual and auditory areas in both directions, we had anticipated that weak auditory stimuli would interact with visual stimuli just as strongly as weak visual stimuli interacted with auditory stimuli. However, our data contradict this hypothesis, and reveal an absence of weak auditory influences on the task with the visual stimuli. Differences in task and stimuli between prior studies and ours may have played a role in these divergent results. These previous studies presented their stimuli more centrally [foveal and parafoveal between 0 and 8° (Shams et al., 2002; Bolognini et al., 2010), 10° (Chen et al., 2017) in humans] or at a maximal peripheral location of 20° for monkeys (Wang et al., 2008). The cited prior studies also differed from ours in many other aspects (e.g., higher intensity stimuli, different tasks, different auditory, and visual features). Therefore, it is difficult to determine what underlies the absence of an auditory influence on the visual stimuli in our study. A hypothesis we currently entertain is that the auditory features presented during the visual task in the far periphery may not have engaged enough attention to be effective in influencing visual processing. Indeed, most of the participants reported being oblivious of the low-intensity auditory stimuli, supporting this hypothesis.

To summarize, in our paradigm studying audiovisual interactions in far periphery, weak visual stimuli influenced the response times in an auditory modulation task (with facilitation and degradation depending on specific temporal conditions), but a reverse auditory influence on the visual task was not observed. Due to a programming error, eye movements were only recorded before stimulus presentation and at the response. However, fixation samples at response time in each trial strongly support that the participants fixated accurately (in 96% of the ∼100,000 trials fixation samples fell within 2.5° of the fixation center, see section “Materials and Methods”). Although our conclusions would have been stronger without our programming error, the fixation data we do have make it unlikely that the observed asymmetrical nature of multisensory interaction would be due to a confounding effect of inaccurate fixation. The observed visual-to-auditory influences only occurred for appropriate phase differences between the modulated AV stimuli. Our data support a potential role of direct interactions between early visual and auditory areas through manipulation of AV synchrony (Lakatos et al., 2007), which remains to be tested in future neurophysiological and neuroimaging studies. The involvement of early sensory regions in multisensory processing of stimuli at peripheral locations does not exclude a major role for higher-order cortices. Multisensory integration is a multifaceted process, and higher-order cortices are likely involved in among others directing attention, object recognition and cross-trial effects. Hence, our findings are compatible with a view in which both the early auditory and visual cortices as well as higher-order auditory and visual cortex contribute to multisensory integration (Ghazanfar and Schroeder, 2006). This research extends the behavioral evidence of the importance of cross-sensory temporal cues for auditory processing (Besle et al., 2008; Doesburg et al., 2008; Stevenson et al., 2010) to the far periphery. By combining temporally and spatially high-resolution neuroimaging techniques, future studies may provide insight into the precise temporal mechanisms and the cortical sites driving cross-modal interactions. This future work may also provide insights into similarities and differences of mechanisms underlying cross-sensory interactions at eccentricities ranging from foveal to far-peripheral space.

## Data Availability Statement

The datasets generated for this study can be found in online repositories. The names of the repository/repositories and accession number(s) can be found below: https://github.com/macsbio/audiovisual-periphery.

## Ethics Statement

The studies involving human participants were reviewed and approved by Ethics Review Committee of the Faculty of Psychology and Neuroscience at Maastricht University. The patients/participants provided their written informed consent to participate in this study.

## Author Contributions

All authors designed the study. IZ conducted the experiments. IZ, MM, and AL-C analyzed the data. All authors interpreted the data. IZ wrote the manuscript. The manuscript was reviewed and edited by all authors.

## Conflict of Interest

The authors declare that the research was conducted in the absence of any commercial or financial relationships that could be construed as a potential conflict of interest.

## Publisher’s Note

All claims expressed in this article are solely those of the authors and do not necessarily represent those of their affiliated organizations, or those of the publisher, the editors and the reviewers. Any product that may be evaluated in this article, or claim that may be made by its manufacturer, is not guaranteed or endorsed by the publisher.
