# Supplementary material for: Audiovisual Interactions Among Near-Threshold Oscillating Stimuli in the Far Periphery Are Phase-Dependent
Source: Front Hum Neurosci. 2021 Aug 30;15:642341. doi: 10.3389/fnhum.2021.642341 (PMC8435850; doi:10.3389/fnhum.2021.642341)
Supplement: Supplementary file 1 [file Table_1.docx]

Audiovisual Interactions among Near-threshold Oscillating Stimuli in the Far Periphery are Phase-dependent

**Isma Zulfiqar^1,2*^, Michelle Moerel^1-3^, Agustin Lage-Castellanos^1^, Elia Formisano^1-3^, and Peter De Weerd^1-2^**

^1^Department of Cognitive Neuroscience, Faculty of Psychology and Neuroscience, Maastricht University, Maastricht, 6200 MD the Netherlands, ^2^Maastricht Centre for Systems Biology, Maastricht University, Maastricht, 6200 MD, the Netherlands, ^3^Maastricht Brain Imaging Center (MBIC), 6200 MD Maastricht, the Netherlands

*** Correspondence:**Isma Zulfiqar (isma.zulfiqar@maastrichtuniversity.nl)

*Supplementary Material*

# Supplementary Data

## Congruency does not influence Auditory and Visual Modulation Detection Thresholds

We first explored the effects of the congruency of AV streams (modulated and static) and the phase relation between modulated AV stimuli on modulation detection thresholds. Supplementary Figure 4 shows auditory (A-C) and visual (D-F) modulation detection thresholds obtained in the unisensory condition, as well as those obtained in the audiovisual congruent and incongruent conditions for each phase-condition. No effect of (in)congruence was observed on either the auditory or the visual detection thresholds for any phase condition as shown by the following statistical analysis.

Auditory detection thresholds are shown in Supplementary Figure 4 (A-C). A mixed ANOVA showed neither significant main effects nor interactions among between-subject factor “Phase condition” (3 levels: φ_A=V_ – φ_A>V_ – φ_V>A_), and within-subject factors “Sensory condition” (3 levels: unisensory – congruent – incongruent) and “Intensity” (65% – 55%).

Visual detection thresholds are shown in Supplementary Figure 4 (D-F). A nearly significant 3-way interaction (*F*(4,48) = 2.48, *p* = 0.056) was found between “Phase condition” (3 levels: φ_A=V_ – φ_A>V_ – φ_V>A_), “Sensory condition” (3 levels: unisensory – congruent – incongruent), and Intensity (2 levels: 65% – 55%). All other interactions and main effects failed to reach significance.

## Detailed statistical analysis of response times during Auditory task

A mixed four-way ANOVA analysis of between-subject factor: “Phase condition” (levels: φ_A=V_ – φ_A>V_ – φ_V>A_) and the three within-subject factors “Auditory stimulus” (levels: modulated – static), “Visual influence” (levels: modulated – static – none) and “Intensity” of the visual influence (levels: 55% – 65%) showed a significant four-way interaction (*F*(4,48) = 2.957, *p* = 0.029). The interaction was analyzed for each level of phase condition for further analysis (Supplementary Figure 5). As concluded by following analysis, the effects across “Intensity” levels were not significantly different across phase conditions and thus the observations were simplified in the main text by combining both levels.

For phase condition φ_A=V_, there was no significant three-way interaction between factors “Auditory stimulus”, “Visual influence” and “Intensity”. The two-way interactions and main effects also failed to reach significance.

For phase condition φ_A>V_, the three-way interaction between “Auditory stimulus”, “Visual influence” and “Intensity” was significant (*F_0.016_*(2,48) = 6.267, *p* = 0.003). This interaction was explored for modulated and static auditory stimuli separately. For modulated auditory stimuli, there was no significant interaction between factors “Visual influence” and “Intensity”. The main effect of “Visual influence, however, was significant (*F*(2,16) = 12.7411, *p* < 0.001, α = 0.008). Post hoc comparisons showed that modulated visual influence sped up response times for static auditory stimuli, while static visual influence slowed down the responses (modulated vs static *t*(8) = -4.31, *p*[corrected] = 0.007, modulated vs none *t*(8) = -1.78, *p*[corrected] = 0.337, static vs none *t*(8) = 3.54, *p*[corrected] = 0.02). For static auditory stimuli, the interaction between factors “Visual influence” and “Intensity” was significant (*F_0.008_*(2,48) = 5.38, *p* = 0.007) and was further explored for the two levels of Intensity (55% and 65%). At 55% intensity, there was a significant effect of “Visual influence” (*F_0.003_*(2,16) = 14.366, *p* < 0.001; modulated vs static: *t*(8) = -4.14, *p*[corrected] = 0.009; modulated vs none: *t*(8) = -5.217, *p*[corrected] = 0.002, static vs none *t*(8) = 0.232, *p*[corrected] > 0.999). “Visual influence” also significantly affected the response times at 65% intensity (*F_0.003_*(2,16) = 19.08, *p* < 0.001; modulated vs static *t*(8) = -5.178, *p*[corrected] = 0.002; modulated vs none *t*(8) = -4.32, *p*[corrected] = 0.007; static vs none *t*(8) = 2.87, *p*[corrected] = 0.062). Overall, the presence of a modulated visual influence improved the response times for static sounds irrespective of its intensity.

For phase condition φ_V>A,_ there was a significant three-way interaction between “Auditory stimulus”, “Visual influence” and “Intensity” (*F_0.016_*(2,48) = 6.64, *p* = 0.002). This interaction was broken down for each level of “Auditory stimulus”. For modulated auditory stimuli, the main effect of “Visual influence” was significant (*F_0.008_*(2,16) = 58.99, *p* < 0.001; modulated vs static *t*(8) = -7.698, *p*[corrected] < 0.001; modulated vs none *t*(8) = -8.55, *p*[corrected] < 0.001; static vs none *t*(8) = 5.778, *p*[corrected] = 0.001). The two-way interaction between “Visual influence” and “intensity” and the main effect of “Intensity” was insignificant. In case of static auditory stimuli, the interaction between “Visual influence” and intensity was significant (*F_0.008_*(2,48) = 10.174, *p* < 0.001). This interaction was further explored for each level of “Intensity”. At 55% intensity, “Visual influence” had a significant effect on response times of static sounds (*F_0.003_*(2,16) = 18.5, *p* < 0.001; modulated vs static *t*(8) = -3.27, *p*[corrected] = 0.03; modulated vs none *t*(8) = -11.89, *p*[corrected] < 0.001; static vs none *t*(8) = -1.468, *p*[corrected] = 0.54). The “Visual influence” at 65% also showed significant effects (*F_0.003_*(2,16) = 10.67, *p* = 0.001; modulated vs static *t*(8) = -3.40, *p*[corrected] = 0.02; modulated vs none *t*(8) = -3.86, *p*[corrected] = 0.01; static vs none *t*(8) = 1.33, *p*[corrected] = 0.65).

# Supplementary Figures


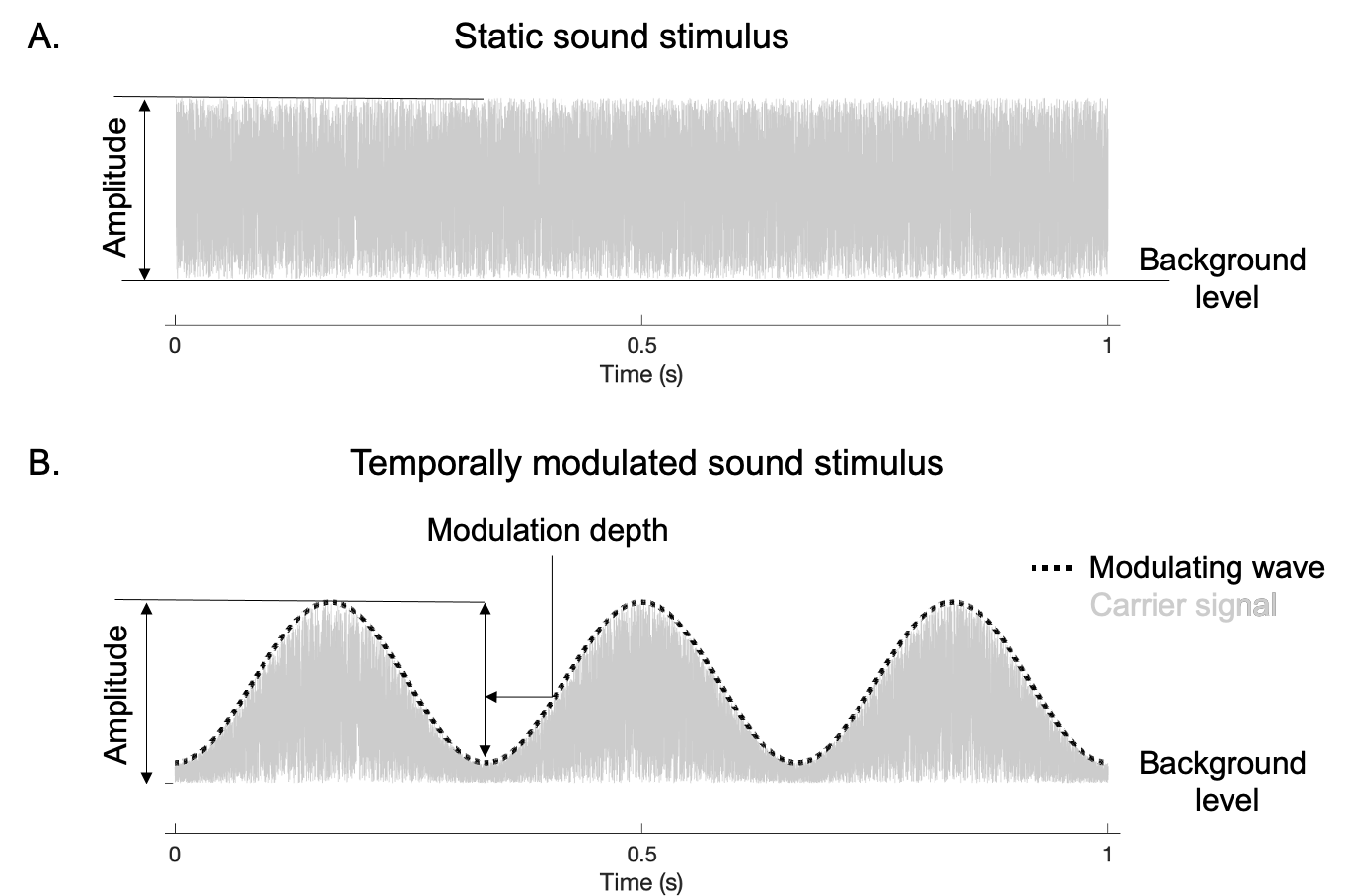


**Supplementary Figure 1: Stimulus dynamics of the sound stimulus.** (A) The static auditory stimulus. This stimulus comprised a white noise burst at a certain amplitude. (B) The modulated auditory signal. This signal was generated by temporally modulating the white noise signal shown in panel A by a 3 Hz modulating wave (black dotted line) with a modulation depth of 80%. Here we have shown a modulating wave without phase-shift at the onset. The phase-shifts of the other modulating waves used in the experiment are shown in Figure 1A. The amplitude of either sound (modulated and static) was varied to adjust (increase/decrease) the sound pressure level in the staircases to ongoing performance. In a similar manner, the static visual stimulus (vertical grating) consisted of a certain contrast (akin to the amplitude shown here). In the modulated visual stimulus, the contrast was temporally modulated over time at 3 Hz with a modulation depth of 80%, as shown in panel B for the auditory stimulus. Michelson contrast was computed using the maximum and minimum luminance levels of the visual grating, and was used to quantify stimuli (modulated and static) of the staircases. All stimuli were 1s in duration. Note that in the staircase, each level (or step) corresponded to a specific amplitude of the static stimulus and it’s corresponding temporally modulated stimulus. In summary, the signal strength of the stimuli was made dependent on performance, while always keeping the modulation depth in temporally modulated stimuli constant at 80% of the maximal modulation depth. Thus, in the modulation detection task, participants had to report whether they detected the 80% modulation or not, during staircase threshold measurements in which the signal amplitude was made dependent on performance.


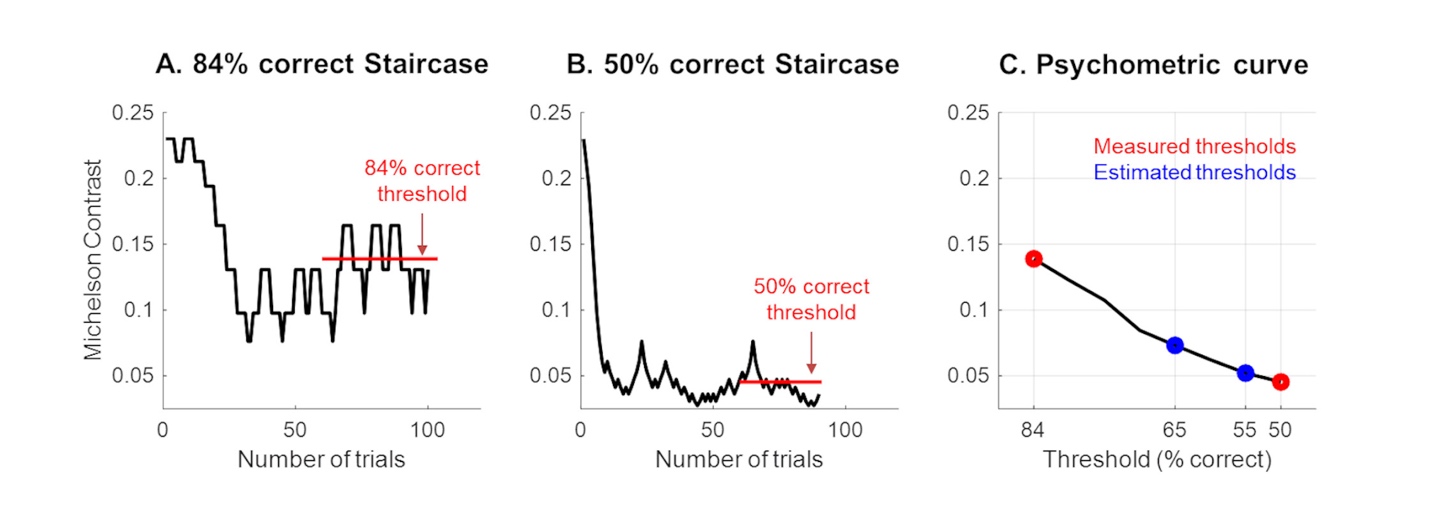


**Supplementary Figure 2:** Estimation of unisensory modulation detection thresholds for a single participant executing the visual task. (A) Measurements (black line) in an 84% correct detection threshold staircase (4 correct responses: contrast down, 1 incorrect response: contrast up). The final threshold is shown by the red line and is computed as the mean of the last 10 reversal points. (B) The 50% detection threshold measurements (black line, 1 correct response: contrast down, 1 incorrect response: contrast up) are shown, with the red line indicating chance detection level (the mean of the last 10 reversal points). (C) The measured 84% and 50% correct contrast thresholds (in red) are used to compute the 65% and 55% correct contrast thresholds (in blue) used in the multisensory conditions. Specifically, the 65% and 55% detection steps are estimated by z-scoring the contrast steps between the 84% and 50% measurements, and then interpolating the intermediate steps (from 50% to 84%). Note that in the staircase, each level (or step) corresponded to a specific amplitude of the static stimulus and its corresponding temporally modulated stimulus (see Supplementary Figure 1). Overall, the signal strength of the stimuli was made dependent on performance, while always keeping the modulation depth in temporally modulated stimuli constant at 80% of the maximal modulation depth. Thus, in the modulation detection task, participants had to report whether they detected the 80% modulation or not, during staircase threshold measurements in which the signal amplitude was made dependent on performance.


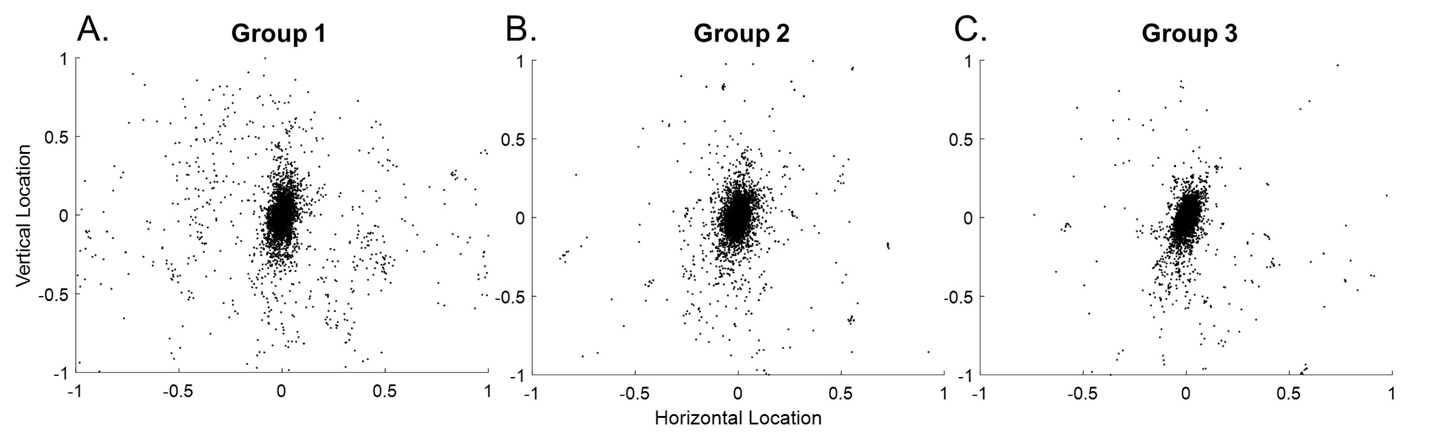


**Supplementary Figure 3: Eye location at the instance of response for all trials shown for the three participant groups.** The axes indicate the location along the screen, where the participants were required to fixate at the center (0,0) and the visual stimulus was presented at the farthest right location along the azimuth. Participants across groups were fixating close to the fixation center in the vast majority of the trials and showed no particular bias towards the stimulus location.
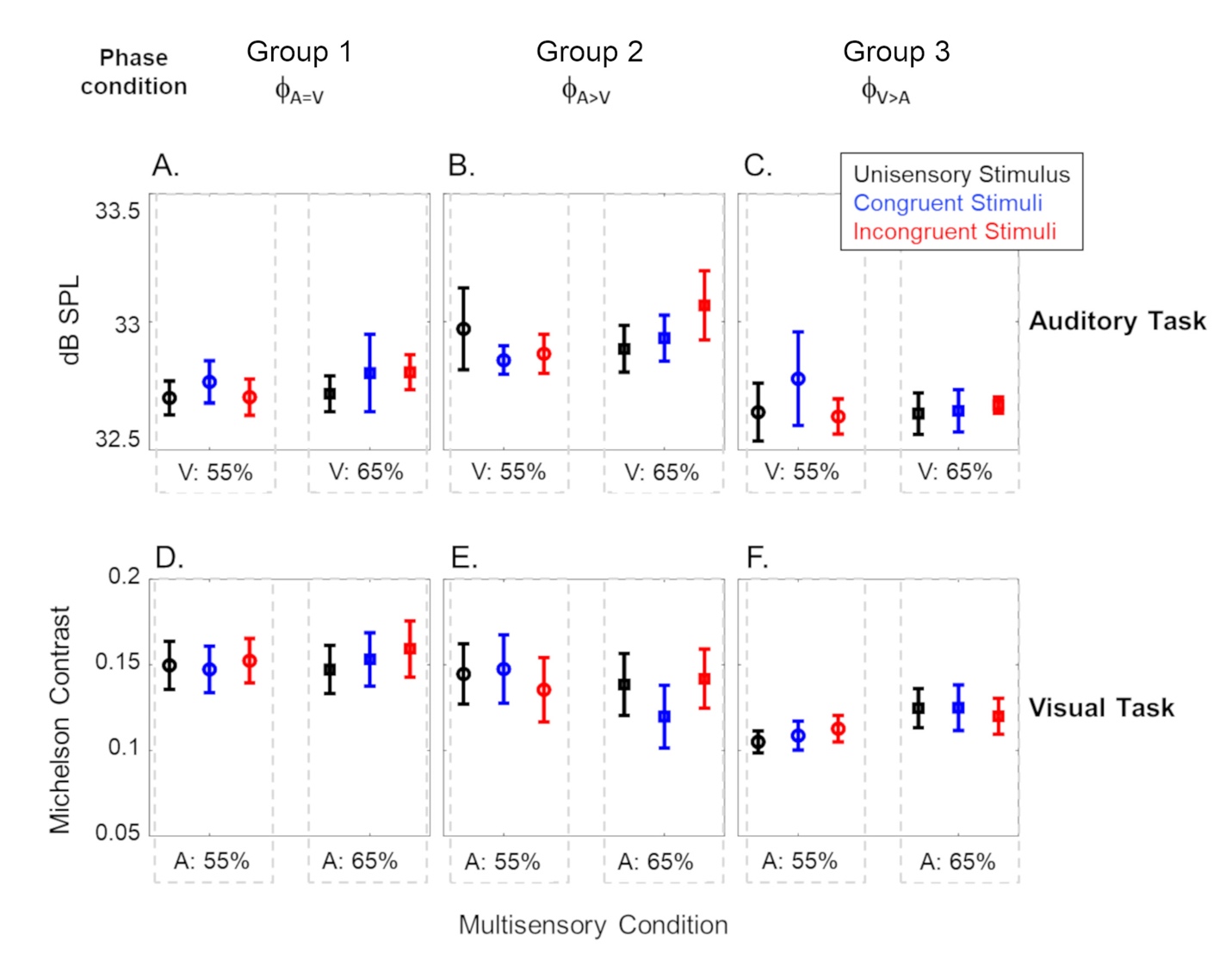


**Supplementary Figure 4.** Auditory and visual modulation detection thresholds. Auditory detection thresholds are shown in (A-C) for all phase conditions (φ_A=V,_ φ_A>V,_ φ_V>A_ respectively). The SPL values are shown for the peak intensity of the stimuli. Visual detection thresholds are shown in (D-F) for all phase conditions. The detection thresholds for all unisensory (black), congruent (blue) and incongruent (red) conditions are plotted for two intensities of the unattended modality (at estimated 55% and 65% detection threshold). Error bars represent ± 1 SEM.


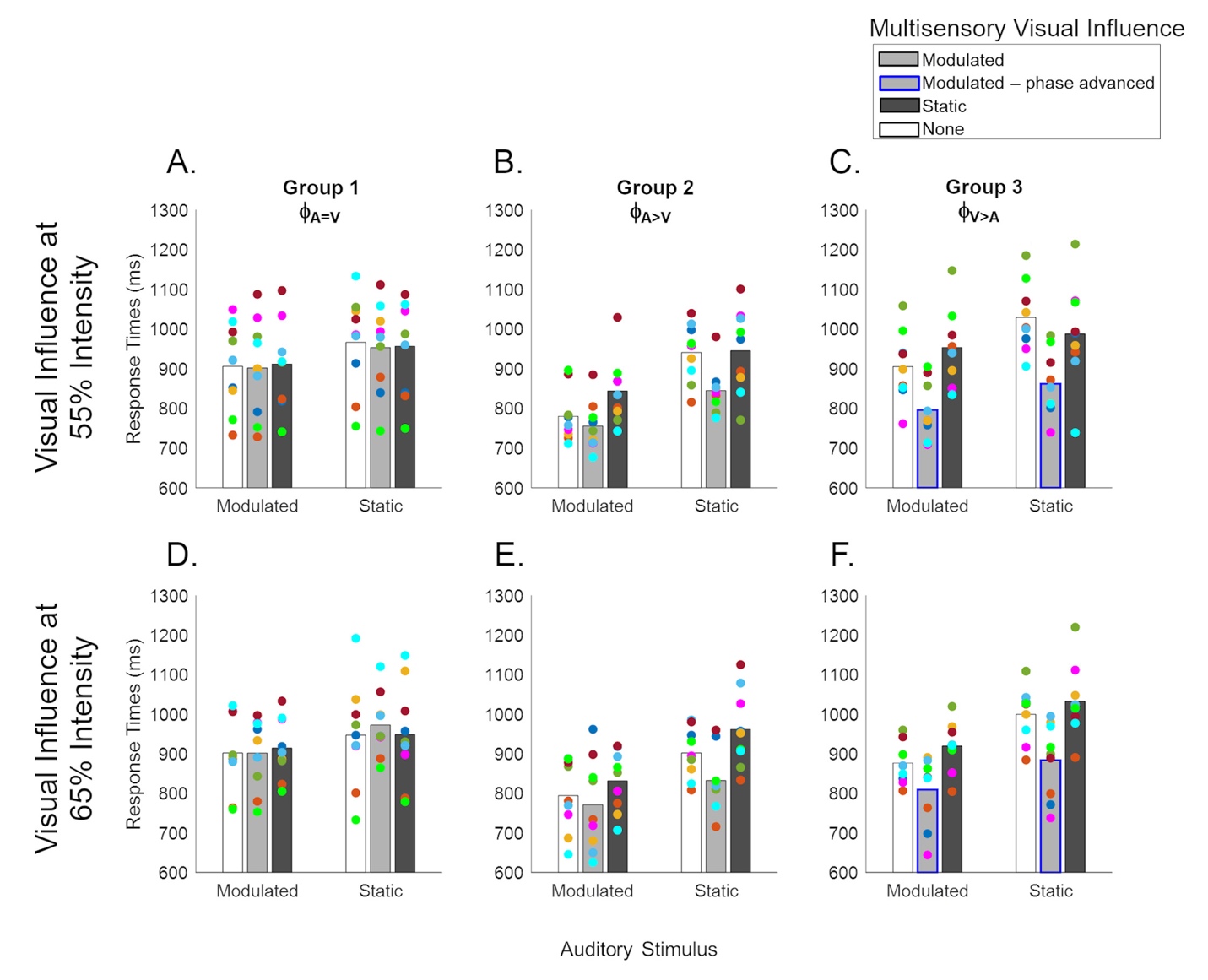


**Supplementary Figure 5**: Response times during the auditory modulation detection task with visual influences. The figure shows the interaction of “Auditory stimulus” and “Visual influence” plotted separately for the three phase conditions φ_A=V_ (Group 1), φ_A>V_ (Group 2) and φ_V>A_ (Group 3), with “Intensity” of visual influence at 55% (A-C) and 65% (D-F). Light and dark gray bars represent the presence of near-threshold modulated and static visual influences respectively, while white bars show the unisensory condition. Grey bars with a blue outline indicate the phase-advanced visual condition. Individual participant data is shown using colored dots (different groups refer to different participant samples). Bar height reflects the mean of the data.


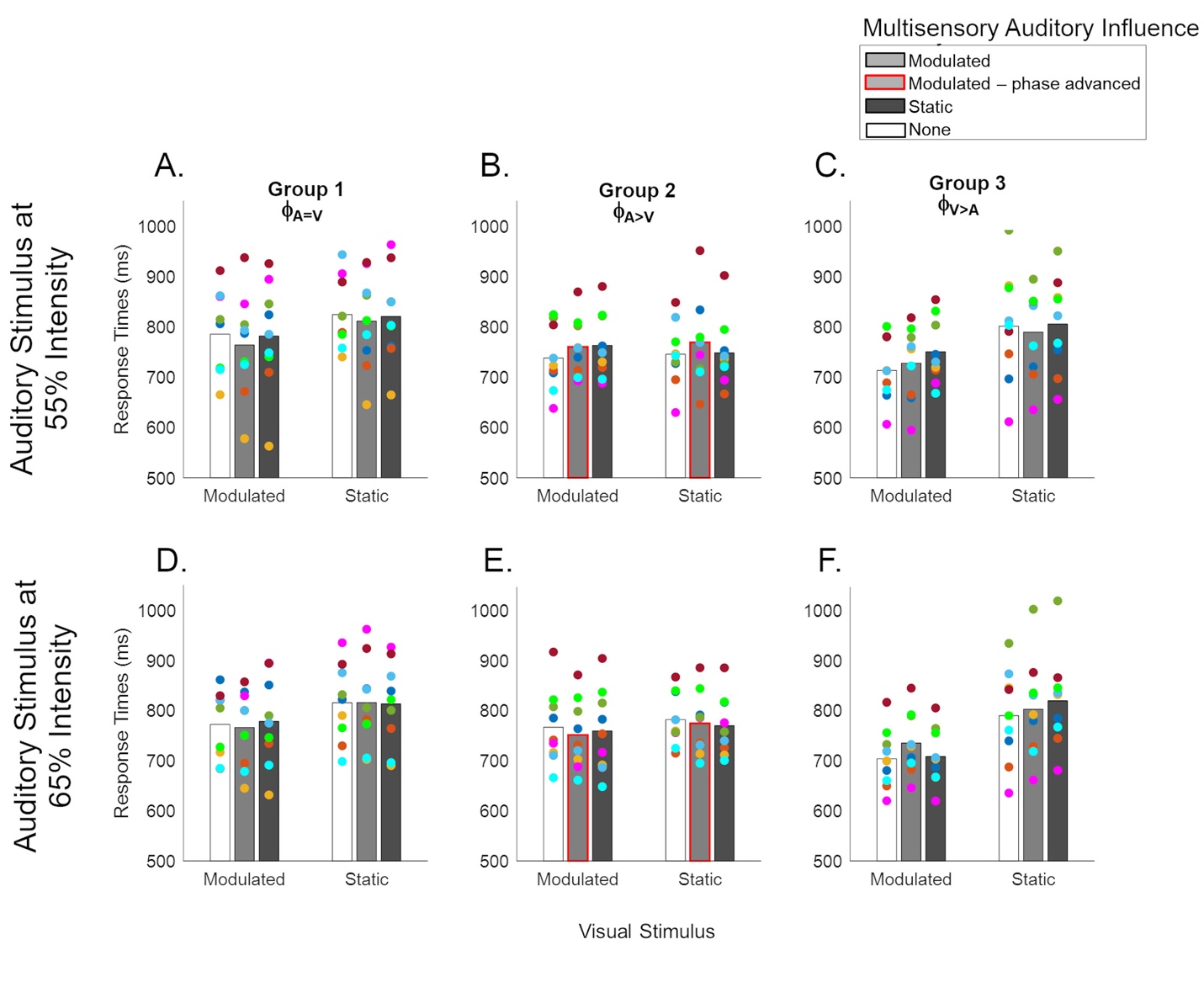


**Supplementary Figure 6**: Response times during the visual modulation detection task with auditory influences. How response times for “Visual stimulus” are affected by “Auditory influence”, is plotted separately for the three phase conditions φ_A=V_ (Group 1), φ_A>V_ (Group 2) and φ_V>A_ (Group 3), with “Intensity” of auditory influence at 55% (A-C) and 65% (D-F). Light and dark gray bars represent the presence of near-threshold modulated and static visual influences respectively, while white bars show the unisensory condition. The grey bars with a red outline indicate the phase-advanced auditory condition. Individual participant data is shown using colored dots (different groups refer to different participant samples). Bar height reflects the mean of the data.
